# Switching Hydrogen Bonding to π-Stacking: The Thiophenol Dimer and Trimer

**DOI:** 10.1021/acs.jpclett.0c03797

**Published:** 2021-01-28

**Authors:** Rizalina Tama Saragi, Marcos Juanes, Cristóbal Pérez, Pablo Pinacho, Denis S. Tikhonov, Walther Caminati, Melanie Schnell, Alberto Lesarri

**Affiliations:** † Departamento de Química Física y Química Inorgánica, Facultad de Ciencias-I.U. CINQUIMA, Universidad de Valladolid, Paseo de Belén, 7, E-47011 Valladolid, Spain; ‡ Deutsches Elektronen-Synchrotron DESY, Notkestraße 85, D-22607 Hamburg, Germany; § Dipartimento di Chimica Giacomo Ciamician, Via Selmi, 2, I-40126 Bologna, Italy; ∥ Institut für Physikalische Chemie, Christian-Albrechts-Universität zu Kiel, Max-Eyth-Str. 1, D-24118 Kiel, Germany

## Abstract

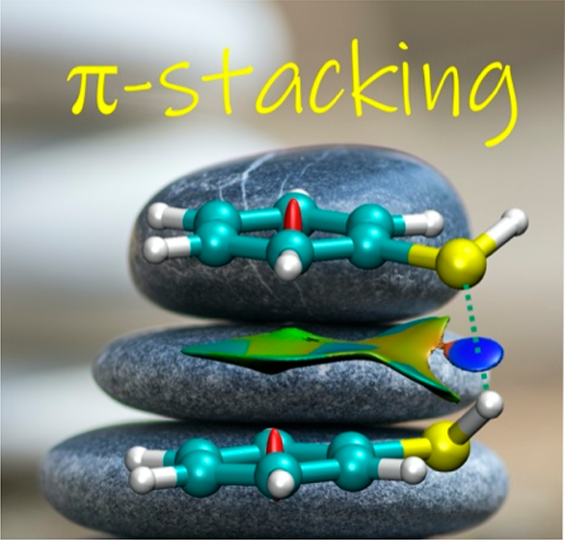

We used jet-cooled broadband rotational spectroscopy to explore the balance between π-stacking and hydrogen-bonding interactions in the self-aggregation of thiophenol. Two different isomers were detected for the thiophenol dimer, revealing dispersion-controlled π-stacked structures anchored by a long S–H···S sulfur hydrogen bond. The weak intermolecular forces allow for noticeable internal dynamics in the dimers, as tunneling splittings are observed for the global minimum. The large-amplitude motion is ascribed to a concerted inversion motion between the two rings, exchanging the roles of the proton donor and acceptor in the thiol groups. The determined torsional barrier of *B*_2_ = 250.3 cm^–1^ is consistent with theoretical predictions (290–502 cm^–1^) and the monomer barrier of 277.1(3) cm^–1^. For the thiophenol trimer, a symmetric top structure was assigned in the spectrum. The results highlight the relevance of substituent effects to modulate π-stacking geometries and the role of the sulfur-centered hydrogen bonds.

π-Stacking forces are fascinating interactions with a misguiding name, leading some authors to dismiss this term.^1,2^ Noncovalent interactions between neutral closed-shell unsaturated organic groups are decisive contributors to biochemical structures, as in DNA/RNA nucleobase stacking or protein folding.^3−5^ In addition, the influence of stacking forces extends to organic and organometallic syntheses,^6^ protein and crystal designs,^7^ host–guest compounds,^8^ catalysis,^9^ materials,^10^ and supramolecular chemistry,^6^ calling for a description at a molecular level.

The polar electrostatic or Hunter-Sanders^11^ model initially ascribed π-stacking to quadrupole–quadrupole interactions (1/*r*^7^ distance dependence). However, more recent computational analyses^1,2^ using energy decomposition attribute the physical origin of the π–π stacking stabilization to dispersion forces (1/*r*^6^ dependence), promoted by the close near-parallel biplanar arrangement. The quadrupolar electrostatic potential actually favors the stacking of saturated rings, but this factor is counterbalanced by a reduced Pauli exchange repulsion for arene–arene stacking. Other calculations have explored the balance between dispersion and electrostatic effects^12−14^ or revealed the connection of dispersion and DNA helicity.^15^ However, since arene stacking stabilization is not based upon direct π-cloud attraction the concept of “π-stacking” should only be used as a positional descriptor.

Experiments on stacking are crucial to validate the increasingly complex theoretical models. In particular, gas-phase experiments are unbiased by perturbing matrix effects and directly comparable to the computational predictions. As an illustrative example, the rotational spectrum of the benzene dimer contributed to the theoretical dispute between the observed T-shape^16−18^ and the alternative parallel geometry.^19^ Most of the gas-phase stacking experiments have used double-resonance IR-UV spectroscopy,^20−24^ but their vibrational signatures are usually of low resolution. Microwave spectroscopy provides accurate structural descriptions through the moments of inertia.^25,26^ However, there are just a few rotational investigations of π-stacking clusters. For clusters of a single benzene ring, the serendipitous observation of the 1,2-difluorobenzene dimer^27^ benefited from the changes in the molecular electrostatic potential due to strongly electronegative substituents, but it took years to realize the correct geometry. For two fused rings, dibenzofuran^28^ and 1-naphthol^29^ exhibit stacking, consistent with the increased stability of larger arene dimers.^1^

Apart from fluorination, other weaker substituent effects,^12,13^ like the fine-tuning of hydrogen bonding, can be explored to switch single-ring dimers from nonstacking into stacking. In the case of phenol, the dimer^30,31^ is controlled by a moderately strong O–H···O hydrogen bond that results in a “hinged” structure intermediate between T or stacked geometries, very sensitive to dispersion contributions.^32^ The dimer of aniline shows the opposite effect, with a (head-to-tail) apolar antiparallel stacking and no N–H···N hydrogen bond between the amino groups (Figure 1).^33^ Here, we explore the replacement of oxygen in phenol by a heavier less-electronegative chalcogen atom like sulfur, proving that it maintains S–H···S hydrogen bonding while simultaneously resulting in a π-stacking homodimer. The work is extended also to the thiophenol trimer, complementing our view on sulfur hydrogen bonding^34−37^ and allowing comparisons with the phenol^31^ and aniline^38^ trimers.

**Figure 1:**
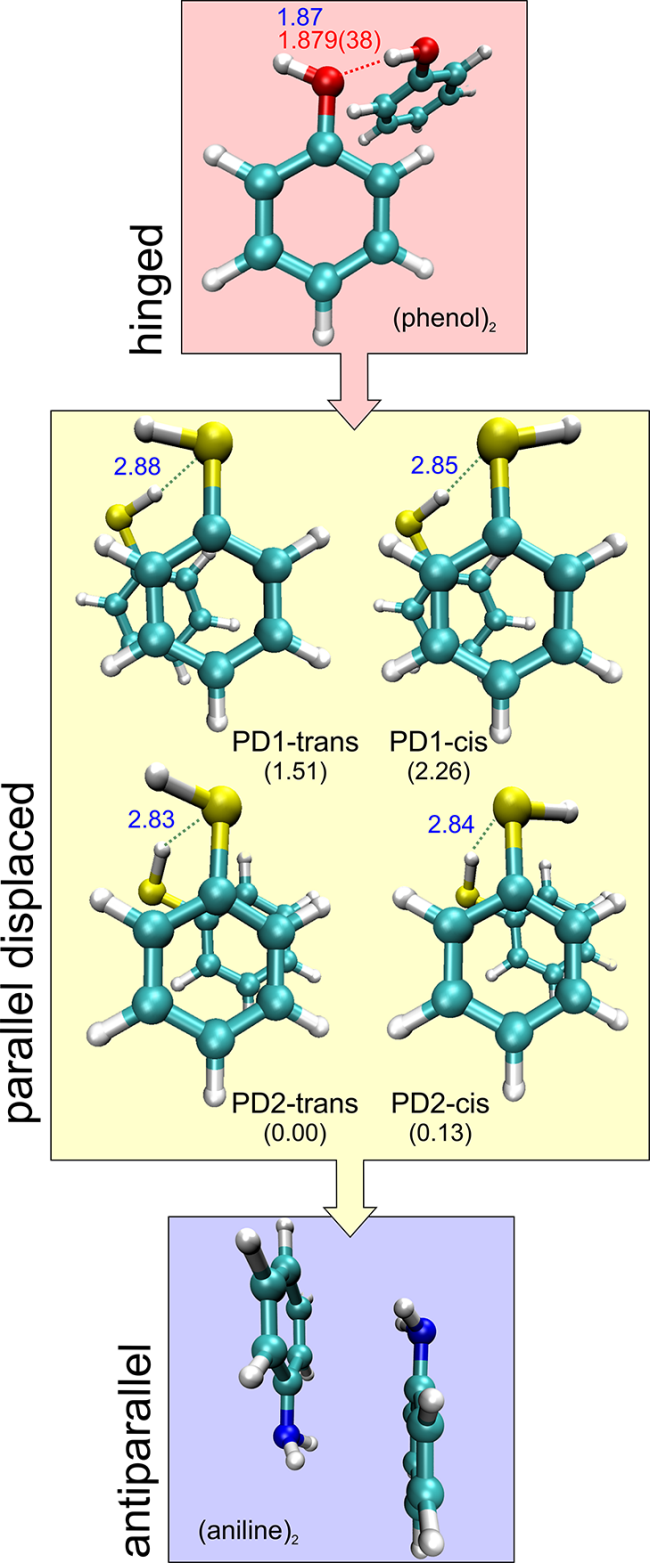
Parallel displaced isomers of the thiophenol dimer compared with the dimers of phenol and aniline. Relative complexation energies (kJ mol^–1^) and S–H···S hydrogen-bond distances (B2PLYP-D3(BJ)/def2-TZVP, Table 1) are given for the thiophenol dimer.

The experiment was assisted by several computational models described in the Supporting Information. We present results based on four density functional theory (DFT) methods, including hybrid (B3LYP, ωB97XD, PBEh-3c) and double-hybrid (B2PLYP) functionals with empirical dispersion corrections.^39^ The B3LYP-D3(BJ) dimer calculations of Table S1, Supporting Information converged to eight structures, with four isomers at electronic energies below 1.4 kJ mol^–1^ and four additional species in the 2–5 kJ mol^–1^ range. The four most stable structures were reoptimized with B2PLYP-D3(BJ) (Table 1) and ωB97XD (Table S2, Supporting Information) to check the computational consistency. For the trimer, B3LYP-D3(BJ) predicted two practically isoenergetic isomers (Table S3, Supporting Information), while six other structures were found at electronic energies below 5 kJ mol^–1^. The two most stable trimer isomers were similarly reoptimized with B2PLYP-D3(BJ) and ωB97XD, as summarized in Tables 2 and S4. All reported species are local minima at their calculation level.

**Table 1:** Rotational Parameters for the Two Isomers of the Thiophenol Dimer

	experiment			theory^a^			
	Isomer I	Isomer II^b^					
		v = 0	v = 1	PD1-*trans*	PD1-*cis*	PD2-*cis*	PD2-*trans*
*A*,^c^ MHz	662.748 50(27)^d^	626.720 05(70)	626.719 15(70)	693.6	690.4	628.4	629.7
*B*, MHz	499.492 41(20)	511.484 22(83)	511.482 95(83)	496.3	496.9	527.6	530.3
*C*, MHz	338.596 68(19)	422.945 94(94)	422.903 05(91)	347.3	348.9	435.8	435.6
κ	–0.01	–0.13		–0.14	–0.13	–0.05	–0.02
Δ*_J_*, kHz	0.1611(13)	0.1884(99)		0.355	0.329	0.077	0.094
Δ_*JK*_, kHz	28.7175(37)	0.090(41)		–0.527	–0.402	0.297	0.225
Δ*_K_*, kHz	–28.7008(36)	–0.199(38)		0.217	0.121	–0.312	–0.253
δ*_J_*, kHz	0.051 85(50)	–0.0276(47)		0.041	0.030	–0.024	–0.022
δ*_K_*, kHz	14.1665(20)	0.330(28)		0.054	0.150	0.495	0.446
Δ*E*_10_, MHz		8.8698(51)					
*N*^e^	145	139					
σ, kHz	7.6	19.8					
|μ_a_|, Debye^f^	not detected	not detected		0.1	0.5	0.0	0.8
|μ_b_|, Debye	detected	detected		1.5	2.1	1.6	1.1
|μ_c_|, Debye	not detected	detected		0.4	0.9	1.1	0.2
Δ*E*,^g^ kJ mol^–1^				0.85	1.56	0.00	0.42
Δ*G*_100 K_, kJ mol^–1^				0.03	0.73	0.00	0.42
Δ*G*_298 K_, kJ mol^–1^				0.00	0.54	1.87	2.42
Δ*E*_c_, kJ mol^–1^				–25.77	–25.02	–27.15	–27.28
*r*(S–H *···*S), Å				2.879	2.846	2.843	2.830
*∠*(S–H*···*S), deg				138.9	140.8	134.5	134.0

a B2PLYP-D3(BJ)/def2-TVZP predictions, see the Supporting Information for B3LYP-D3(BJ) and ωB97XD/cc-pVTZ values.

b Torsional substates denoted v = 0 and 1.

c Rotational constants (*A*, *B*, *C*), Ray’s asymmetry parameter (κ = (2*B* – *A* – *C*)/(*A* – *C*)), Watson’s *A*-reduction centrifugal distortion constants (Δ*_J_*, Δ*_JK_*, Δ*_K_*, δ*_J_*, δ*_K_*) and torsional energy diference (Δ*E*_10_).

d Standard errors in units of the last digit.

e Number of transitions (*N*) and rms deviation (σ) of the fit.

f Electric dipole moments (μ*_α_*, α = *a*, *b*, *c*).

g Relative energies corrected with the zero-point energy (ZPE), Gibbs energy (Δ*G*) at 100 and 298 K (1 atm) and complexation energy (Δ*E*_c_).

**Table 2:** Rotational Parameters for the Thiophenol Trimer

	experiment	theory^a^	
	Isomer 1	UUU	UUD
*A*,^b^ MHz		236.3	243.2
*B*, MHz	233.071 24(18)	236.1	231.8
*C*, MHz		201.1	193.2
κ		0.99	0.54
Δ*_J_*, kHz	0.0123(45)	0.011	0.011
Δ_*JK*_, kHz		0.049	0.017
Δ*_K_*, kHz		–0.055	–0.021
δ*_J_*, kHz		0.000	0.002
δ*_K_*, kHz		–0.072	0.038
|*μ*_a_|, Debye		0.0	0.5
|*μ*_b_|, Debye		0.0	0.3
|*μ*_c_|, Debye		3.1	0.8
*N*	13		
σ, kHz	5.8		
Δ*E*, kJ mol^–1^		0	–0.71
Δ*G*_100 K_, kJ mol^–1^		0	–0.06
Δ*G*_298 K_, kJ mol^–1^		0	–0.06
Δ*E*_c_, kJ mol^–1^		–68.07	–67.82
*r*(S–H*···*S), Å		2.746–2.760	2.758
*∠*(S–H*···*S), deg		154.3–155.6	157.8

a See Table S3 for the notation.

b Parameter definition as in Table 1.

The experimental investigation used supersonic-jet chirped-pulsed Fourier-transform microwave^40^ (CP-FTMW) spectrometers in Valladolid and Hamburg, operating in the region of 2–8 GHz (see the Supporting Information). CP-FTMW spectroscopy is a rotational coherence technique using microwave (MW) linear fast-passage excitation to activate molecular rotational resonances, later recording the time-domain free-induction decay caused by rotational dephasing. The experiment requires fast electronics to tackle the stringently short (μs) excitation times, but the resulting spectra provide full-bandwidth and high dynamical range capabilities, which turn out essential for the analysis of complicated congested spectra.

The observed rotational spectrum in Figures 2 and S1 (Supporting Information) is dominated by intense monomer transitions, previously reported.^41^ Similarly to phenol, thiophenol tunnels between two equivalent planar structures connected by the internal rotation of the thiol group, splitting the ground vibrational state into two torsional-rotation sublevels (Table S5, Supporting Information). However, the internal rotation barrier is much smaller than in phenol, that is, 277.1(3) versus 1213 cm^–1^.^42^ For the thiophenol dimer, two different asymmetric rotors were assigned in the spectrum. Isomer I exhibited only μ_b_ transitions and behaved like a semirigid rotor, so it could be fitted using a Watson’s semirigid rotor Hamiltonian.^43^ Isomer II presented μ_b_ transitions with small (<0.5 MHz) tunneling doublings, indicative of an internal large-amplitude motion (LAM) connecting two symmetry-equivalent structures. A second set of μ_c_ transitions showed larger tunnelling splittings (ca. 17 MHz), nearly independent of the angular momentum quantum number. This fact suggested a μ_c_-inverting motion, so the experimental transitions were fitted to a two-state rovibrational Hamiltonian without Coriolis coupling terms. For the trimer, we found a set of transitions corresponding to the pattern of a symmetric rotor, but we could not resolve the *K* quantum number fine structure. The experimental frequencies of the rotational transitions are collected in Tables S6–S8 (Supporting Information).

**Figure 2:**
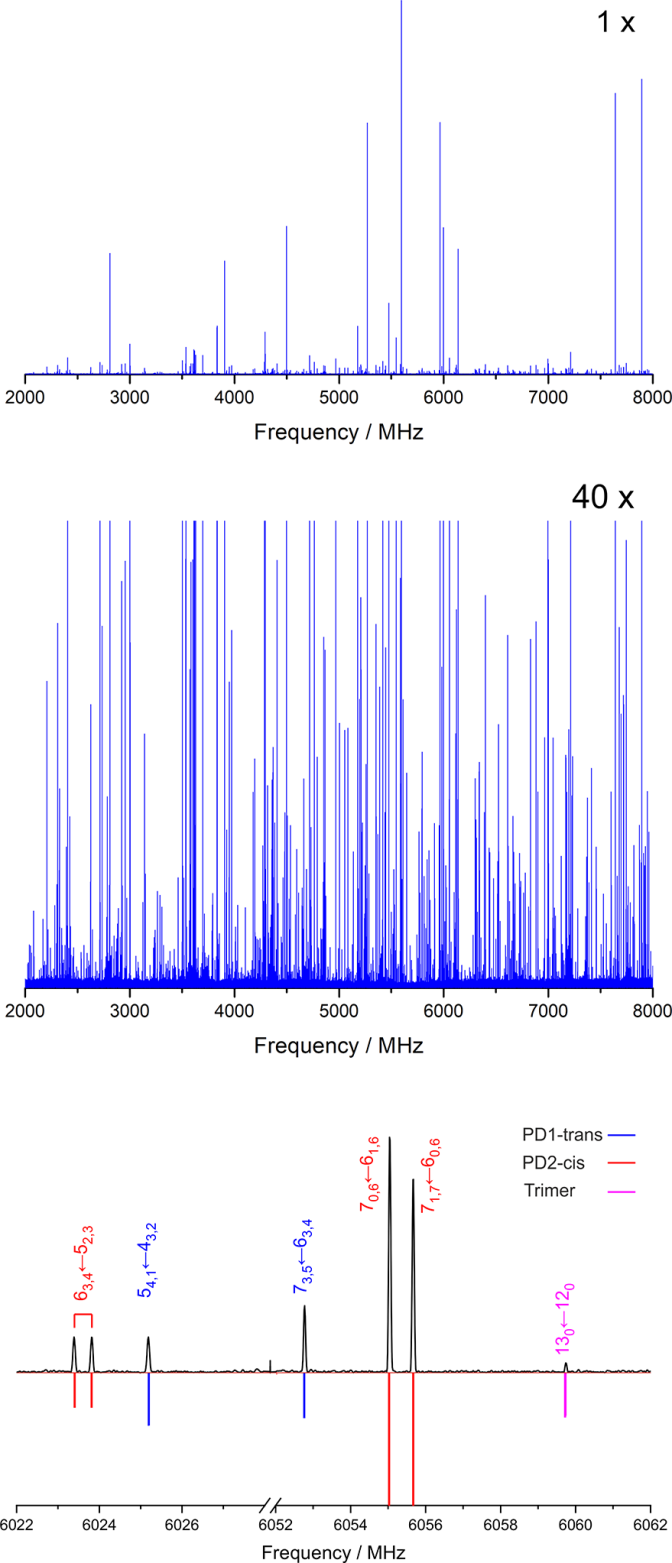
MW spectrum of thiophenol and its aggregates, illustrating typical rotational transitions of the dimer (see also Figure S1).

The comparison between experiment and theory in Tables 1, 2, and S1–S4 (Supporting Information) allowed the identification of the spectral carriers. The predictions suggest parallel displaced (PD) dimer geometries, all sustained by an intermolecular hydrogen bond S–H···S. Two alternative slipped structures are predicted depending on the relative orientation of the phenyl ring with respect to the linking thiol groups, denoted PD1 and PD2 in Figure 1and in the 3D Figures S2 and S3 (Supporting Information). Moreover, for each ring geometry, two isomers arise differing in the parallel (*cis*) or antiparallel (*trans*) orientation of the terminal thiol groups, so four isomers are finally predicted for the dimer. Isomer I was identified as PD1-*trans* based on the rotational constants and dominance of the μ_b_-dipole moment component. Similarly, the μ_c_ spectrum led to the assignment of isomer II as PD2-*cis*. The internal dynamics of PD2-*cis* was attributed to a concerted motion of thiol inversion, which exchanges the proton donor and acceptor moieties (Figure S4 and multimedia, Supporting Information). The inversion barrier was determined from the experimental tunnelling splitting Δ*E*_01_ = 8.8698(51) MHz using Meyer’s flexible model.^44^ Following a consideration of the main structural relaxations associated with the C–S bond (see the Supporting Information) the experiment was reproduced for a potential barrier of *B*_2_ = 250.3 cm^–1^. We compared this barrier with a computational prediction of the torsional potential using DFT and the nudged elastic band algorithm^45^(Supporting Information). The results for three DFT functionals in Table S9 and Figures S5 and S6 (Supporting Information) range from 290 cm^–1^ (ωB97X-D3) to 502 cm^–1^ (B3LYP-D3), giving rise to estimated tunnelling splittings of 13, 4, and 525 MHz (PBEh-3c, B3LYP-D3, and ωB97X-D3, respectively). These calculations confirm a torsional barrier similar to the monomer. A lack of double-minimum symmetry or a ground-state above the barrier prevent tunneling effects for isomer PD1.

The dimer global minimum was identified with a second experiment using argon as carrier gas, checking the possibility of conformational relaxation with more energetic intermolecular jet collisions. The weaker argon spectrum, illustrated in Figures 3 and S7 (Supporting Information), revealed no signals from PD1 and established PD2 as the global minimum. For the thiophenol trimer, the symmetric rotor (UUU in Tables 2 and S3 and Figure S8, Supporting Information), characterized by three consecutive S–H···S hydrogen bonds, can be associated with the observed transitions. No other species could be identified positively, but we do not exclude the presence of other species because of additional unidentified lines.

**Figure 3:**
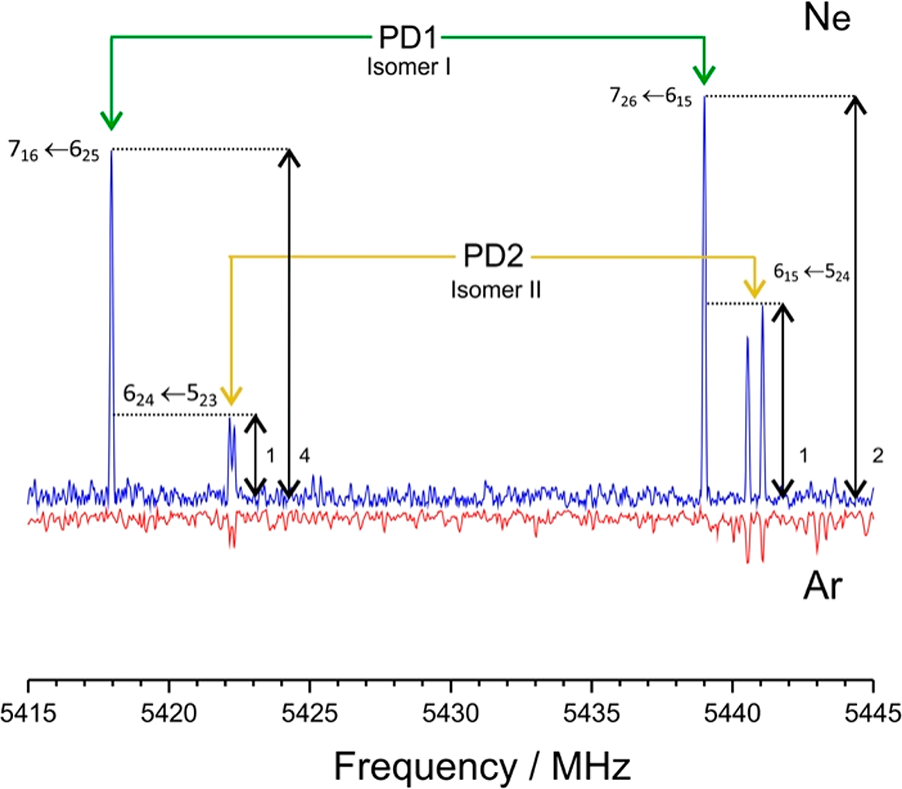
A 30 MHz section of the rotational spectrum of the thiophenol dimer, showing the disappearance of isomer I (PD1) when the neon carrier gas is replaced by argon, enforcing conformational relaxation to the global minimum PD2.

A coherent picture emerges from the present experiment concerning the correlation between thiophenol aggregation and noncovalent interactions. For the thiophenol dimer, the calculations suggest two alternative clustering mechanisms, based either on S–H···S or S–H···π hydrogen bonds. While the relative energies for the first eight isomers are quite close, the preference for a combination of S–H···S hydrogen bond and π-stacking is notorious, offering insight into their structural, energetic, and physical properties. The parallel-displaced global minimum PD2-*cis* exhibits a long hydrogen bond (B2PLYP: *r*(S–H···S) = 2.84 Å) with considerable nonlinearity (∠(S–H···S) = 134.5°). Similar values are presented for PD1 in Figure 1. (Molecular structures are in Tables S10–S13 and in the three-dimensional (3D) Figures S2 and S3, Supporting Information.) This bonding distance is slightly larger than the hydrogen sulfide dimer^46^ prototype (*r*(S–H···S) = 2.778(9) Å) and qualitatively reflects the gradation of hydrogen-bond strength observed in the dimers of H_2_S–H_2_O^47^ (*r*(O–H···S) = 2.597(4) Å), H_2_O–H_2_S^47^ (*r*(S–H···O) = 2.195 Å), and (H_2_O)_2_^48^ (*r*(O–H···O) = 1.951 Å) in Table S14 (Supporting Information). Thiol-alcohol gas-phase hydrogen bonds were also reported for the monohydrates of furfuryl^36^ and thenyl^37^ mercaptan (*r*(S–H···O) = 2.22–2.44 Å; *r*(O–H···S) = 2.43–2.58 Å), but the experimental investigations of gas-phase hydrogen bonds between thiols are still scarce.^34,35^ Protein crystal contacts between the cysteine thiol and the sulfur atom in methionine or cysteine have shorter average values of *r*(S–H···S) = 2.55(47) Å.^49^

The π-stacking geometry of the thiophenol dimers is characterized by the distance between centroids (*d*) and the angle between aromatic planes (ϕ). The interplanar distances, shorter for PD2 (B2PLYP: *d*(PD2) = 3.41–3.42 Å < *d*(PD1) = 3.76–3.77 Å), and the ring orientations (B2PLYP: ϕ (PD2) = 2.9°–4.4° < ϕ (PD1) = 9.2°–10.2°) nicely match previous structural surveys of protein–ligand interactions between aromatic groups, confirming a common binding pattern.^50^ For the trimer, the final geometry in Figure S8 and Table S15 (Supporting Information) balances both S–H···S and C–H···π interactions, as in phenol and aniline, with a hydrogen-bond distance of *r*(S–H···S) = 2.75 Å (B2PLYP).

The physical origin of the noncovalent interactions was modeled by a topological analysis of the reduced electronic density gradient *s* and energy decomposition. The noncovalent interactions (NCI) plots in Figures 4, 5, and S9 (Supporting Information) indicate a confluence of the S–H···S hydrogen bond and delocalized interaction regions between the aromatic rings, consistent with the observed geometries. A binding energy decomposition using Symmetry-Adapted Perturbation Theory SAPT 2+(3) in Figure 6 and Table S16 (Supporting Information) offers comparison with phenol and aniline. The SAPT 2+(3) binding energy of the thiophenol dimer (PD1: −25.9 kJ mol^–1^; PD2: −26.9 kJ mol^–1^) is only 1–2 kJ mol^–1^ smaller than in the phenol dimer (−27.6 kJ mol^–1^). However, it shows a much larger dispersion component than in phenol, accounting for 185.2% (PD1) or 198.5% (PD2) of the total binding energy, close to the contribution in the van der Waals dimer of pyridine-methane (208.1%). In parallel, the electrostatic contribution in thiophenol is reduced to 96.4% (PD1) and 97.4% (PD2) of the binding energy, compared to 151.3% in the phenol dimer or 57.5% in pyridine-methane.

**Figure 4:**
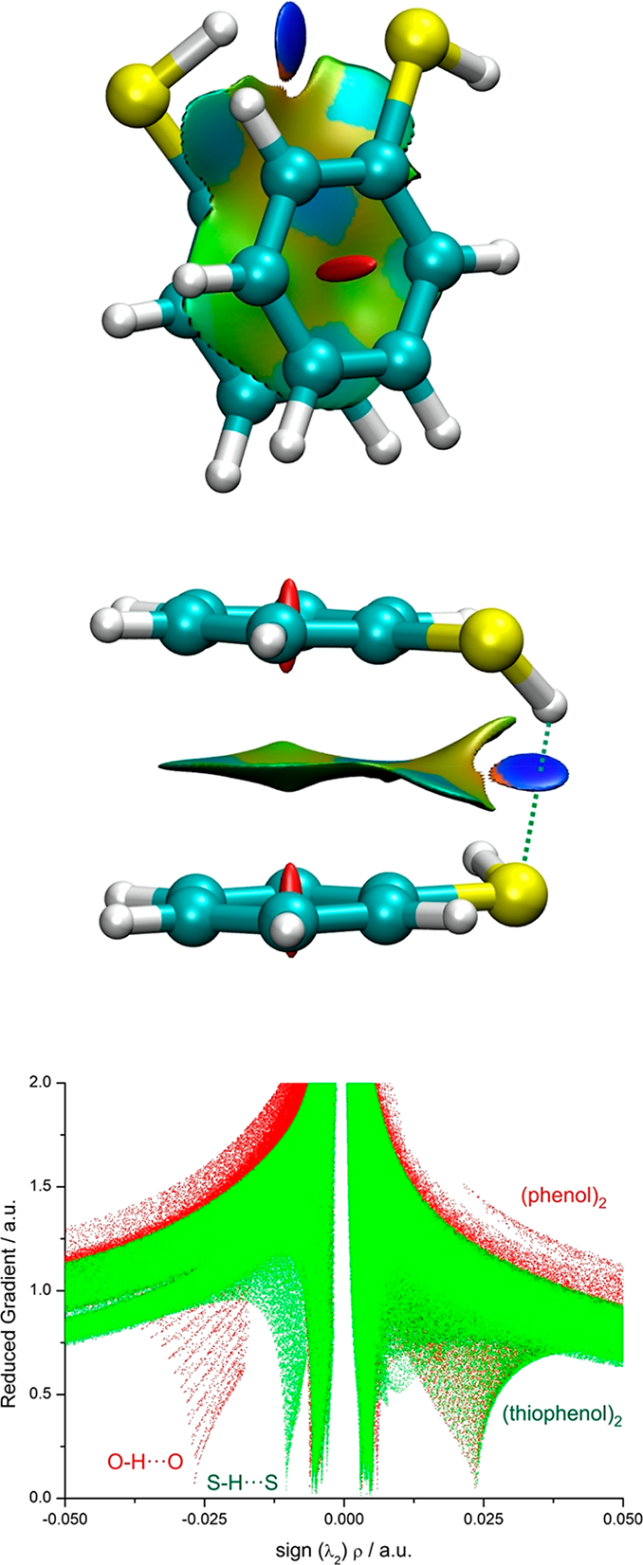
Mapping of NCIs in the most stable (PD2-*cis*) dimer structure of the thiophenol dimer and comparison of the reduced gradient with the phenol dimer.

**Figure 5:**
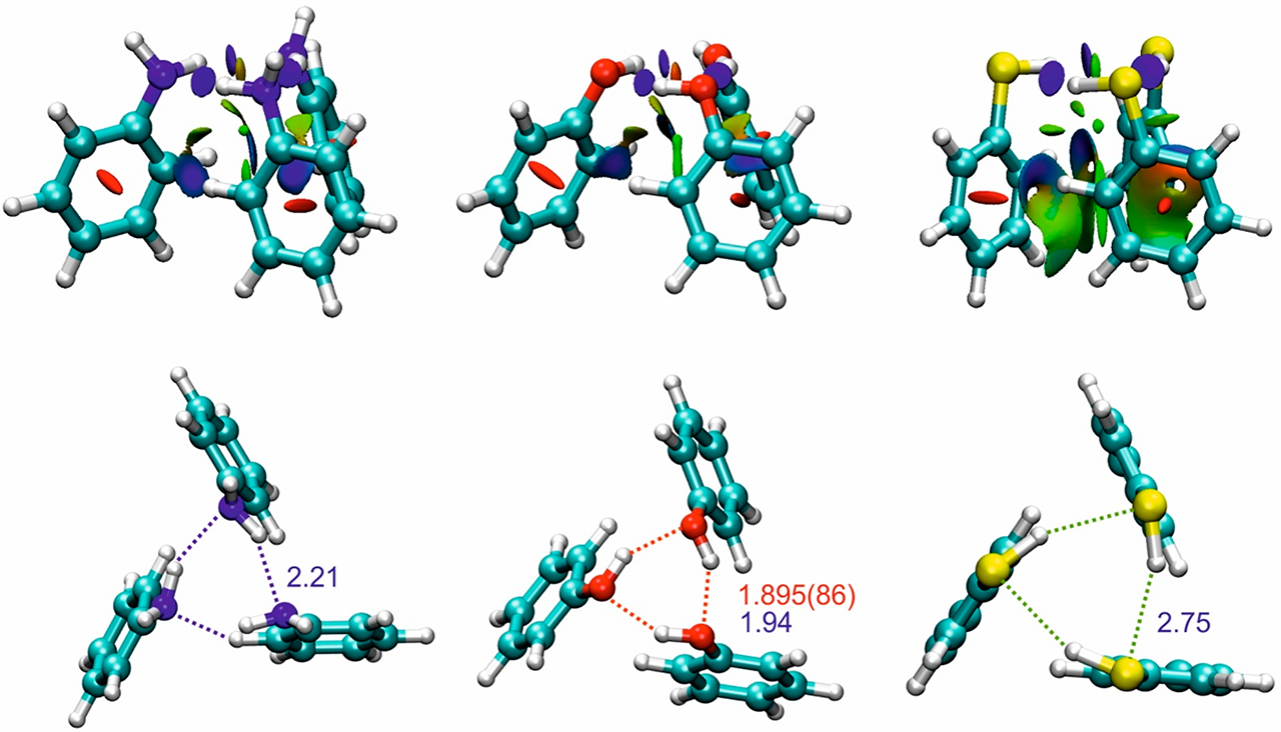
NCI plots for the *C*_3_-symmetric structures of the trimers of aniline (left), phenol (center), and thiophenol (right, hydrogen-bond distances according to B2PLYP-D3(BJ)).

**Figure 6:**
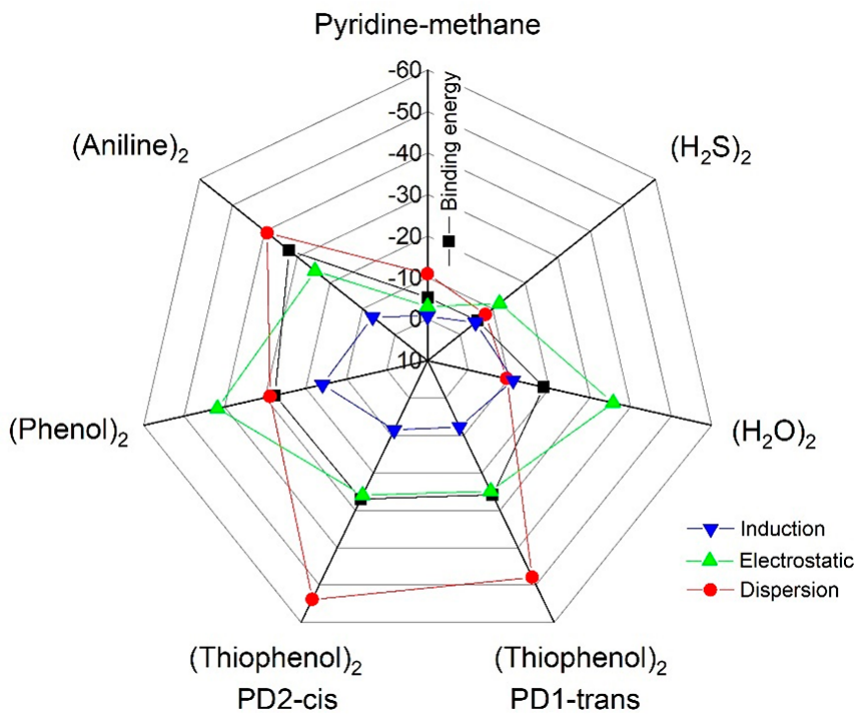
A radar chart showing the SAPT2+(3) binding energy decomposition for the thiophenol dimers (PD1-*trans* and PD2-*cis*) and comparison with the dimers of phenol, aniline, water, hydrogen sulfide, and pyridine-methane reported in Table S16.

In conclusion, chirped-pulse rotational spectroscopy opens new avenues for the investigation of increasingly larger adducts, simultaneously offering a striking comparison with low-resolution IR studies.^51^ We observed two isomers of the thiophenol dimer, confirming two different π-stacking structures assisted by a long S–H···S hydrogen bond. The dimer geometries reveal flexible internal dynamics, as two different geometries are simultaneously detected, and one of the isomers exhibits an internal large-amplitude motion causing spectral doublings. The experiment also provided empirical evidence to contrast the computational models. The three DFT model predictions were comparable in structural terms, with relative deviations from the experimental rotational constants of 0.2–3.5% (ωB97XD), 0.2–4.0% (B3LYP-D3), and 0.3–4.4% (B2PLYP-D3). The ωB97XD/cc-pVTZ binding energies, previously claimed similar to CCSD(T) for aromatic homodimers,^50^ differ less than 1 kJ mol^–1^ from B2PLYP-D3, with B3LYP-D3 giving larger values by 3–4 kJ mol^–1^. The moderate interaction energies and the energy decomposition balance evidence that the thiophenol dimer represents an interesting case of coexistence of electrostatic and dispersion interactions, with the primary S–H···S hydrogen bond acting as a molecular anchor for the positioning of the phenyl rings. The geometry of the trimer maintains the preference for a cooperative hydrogen-bond network as observed in phenol and aniline, but the *C*_3_ symmetry reflects a delicate balance between the hydrogen bond and C–H···π interactions and may not be present in other trimers. The results emphasize the role of substituent effects to modulate π-stacking geometries and the importance of sulfur-centered hydrogen bonds. The connection between gas-phase aggregation processes and the design of supramolecular architectures remains a challenge for future studies.

## Supporting Information Available

Experimental and theoretical methods (PDF)
Animation showing a concerted motion of thiol inversion, which exchanges the proton donor and acceptor moieties (MPG)

## Supplementary Material

jz0c03797_si_001.pdf

jz0c03797_si_002.mpg
