# Supplementary material for: Switching Hydrogen Bonding to π-Stacking: The Thiophenol Dimer and Trimer
Source: J Phys Chem Lett. 2021 Jan 28;12(5):1367–73. doi: 10.1021/acs.jpclett.0c03797 (PMC8812119; doi:10.1021/acs.jpclett.0c03797)
Supplement: Supplementary file 1 — jz0c03797_si_001.pdf [file jz0c03797_si_001.pdf]

Switching Hydrogen Bonding to π–Stacking: The Thiophenol Dimer and Trimer

SUPPORTING INFORMATION

Rizalina Tama Saragi,† Marcos Juanes,† Cristóbal Pérez,‡ Pablo Pinacho,‡ Denis S. Tikhonov,‡ Walther Caminati,§ Melanie Schnell,‡ Alberto Lesarri†*

†Departamento de Química Física y Química Inorgánica and I.U. CINQUIMA, Universidad de Valladolid, Paseo de Belén, 7, E-47011 Valladolid (Spain)

‡Deutsches Elektronen-Synchrotron DESY, Notkestraße 85, D-22607 Hamburg (Germany) and Christian-Albrechts-Universität zu Kiel, Max-Eyth-Str. 1, D-24118 Kiel (Germany)

§Dipartimento di Chimica Giacomo Ciamician, Via Selmi, 2, I-40126 Bologna (Italy)

Experimental and Computational Methods

1. *Chirp-pulsed Fourier transform microwave spectroscopy*

The experimental investigation was conducted with supersonic-jet chirped-pulsed Fourier-transform microwave (CP-FTMW) spectroscopy,[1,2,3] using instruments in Valladolid[4] and Hamburg[5,6] which covered the region 2-8 GHz. The spectrometers use a direct-digital design following Pate.[7] In this technique a short (1 μs) linear microwave chirp excites the rotational resonances of an expanding molecular jet. The chirp pulse is synthesized digitally with an arbitrary waveform generator, amplified to 20 – 200 W, and broadcasted into the jet through a horn antenna, perpendicular to the vertically moving jet. The jet is generated by a pulsed solenoid valve (Parker, series 9), which injects a gaseous mixture into the expansion chamber through a mm-size circular nozzle. The gas expands against an ultimate vacuum pressure of ca. 10^{-7} hPa produced with an oil diffusion pump and a primary rotary pump. Gas pulses were typically of 500-900 μs duration and contained a small amount of sample highly diluted in a carrier gas. Thiophenol (99%) was obtained commercially and used without further purification. The sample is liquid at room temperature (m.p. -15°C, b.p. 169°C), so it was heated inside a reservoir nozzle (60°C) to increment the vapor pressure (1.4 mm Hg at 20°C), while the stream of the carrier gas (neon or argon at stagnation pressures of 1-3 bar) was flowed over the sample. The pulsed expansion leads to effective cluster generation at the nozzle exit, followed by non-collisional propagation and effective internal cooling (rotational temperatures ca. 2 K). Following the transient excitation the spectrometer records the time-domain free-induction decay caused by rotational dephasing, using a receiving horn antenna, low-noise amplifiers and a digital oscilloscope. Typical acquisition times were of 40 μs. Normally a single gas pulse is probed several times to increase the signal level. The final time-domain data are Fourier transformed using a Kaisser-Bessel window, resulting in linewidths of ca. 100 kHz.[8] Frequency uncertainties for the experimental measurements are estimated as 10 kHz. For the present purposes ca. 1 M spectral averages were acquired at a repetition rate of 5 Hz.

2. *Conformational search and molecular models*

An initial set of starting structures was generated using Molecular Mechanics[9] and conformational searching routines implemented in MacroModel.[10] This calculation was followed by molecular orbital calculations using Kohn-Sham density-functional-theory (DFT),[11] which included full reoptimizations and vibrational frequency calculations. Following previous experiences four DFT methods were

chosen, including hybrid (B3LYP,[12] ωB97X-D[13]), double-hybrid (B2PLYP[14]) and composite (PBEh-3c[15]) functionals. The Becke's three-parameter functional was supplemented with two-body Grimme's D3(BJ) dispersion[16] corrections with Becke-Johnson[17] damping, while Head-Gordon's ωB97X-D includes D2 empirical dispersion.[18] Becke's methods were combined with the Alrich's triple-ζ def2-TZVP basis set,[19] while Dunning's cc-pVTZ[20] was chosen for ωB97X-D. In addition to the rovibrational and electric parameters needed for spectroscopic analysis (Tables 1-2 and S1-S4 below) we calculated the complexation energies relative to the monomers in the cluster geometries (including basis set superposition errors) and Gibbs energies in the range 50 K - 300 K. DFT calculations were implemented in Gaussian16.[21] PBEh-3c was implemented in ORCA.[22]

3. *Topological analysis of the electron density and binding energy decomposition*

The analysis of the non-covalent interactions in the dimer used a calculation of the reduced gradient $s\left(=\frac{1}{2(3\pi^2)^{1/3}}\frac{|\nabla\rho|}{\rho^{4/3}}\right)$ of the electronic density ρ, as implemented in NCIPlot.[23,24] A categorization of the physical forces stabilizing the thiophenol dimer was obtained from a symmetry-adapted perturbation theory[25,26] (SAPT) analysis implemented in PSI4,[27] producing a binding energy decomposition. This perturbative calculation used second-order intramonomer correlation corrections and up to third-order intermonomer dispersion corrections denoted SAPT2+(3)[28] and a double-ζ aug-cc-pVDZ[20] basis set. The interaction energy is decomposed into electrostatic (ΔE_{elec}), inductive (multipole interactions/charge transfer, ΔE_{ind}), exchange repulsion (ΔE_{exch}) and dispersion (ΔE_{disp}) energy terms.

4. *Meyer's flexible model calculations*

The potential energy function describing the concerted internal rotation of the two thiol groups in isomer II (PD2-cis) of the thiophenol dimer was first calculated from the experimental torsional splitting ΔE_{10} (= 8.8698(51), Table 1) using Meyer's flexible model.[29] The determination of two-fold internal rotation barriers using semirigid formalisms has been discussed elsewhere.[30,31] The Meyer's flexible model, which has been extensively used to treat the MW data of molecular complexes,[32] is designed to numerically calculate energies and wavefunctions of vibrational and rotational states for 1- or 2-dimensional (1D, 2D) vibrational problems. This model can be applied to any type of internal motion of any non-linear molecule, with the advantages that it allows for structural relaxation, has no symmetry restrictions, and works efficiently for few mesh points. In the case of a 1D problem, if we consider τ as the parameter to describe the motion (for example the correlated SH internal

rotation coordinate in isomer II), the relaxations of any structural parameter can be taken into account as a function of τ. We assumed the following double minimum potential energy function to be appropriate for our problem:

$$V(\tau) = B_2[1 - (\tau/\tau_0)^2]^2 \quad \text{(M1)}$$

where B_2 is the barrier at τ = 0° and τ_0 is the equilibrium value of the inversion angle. Since the HS-CC dihedral angles at the energy minimum are slightly different for the two thiol groups, we fixed τ_0 to their average value (63°) and adjusted B_2 in order to reproduce the experimental ΔE_{10} splitting. We needed to take into account the structural relaxations of at least three structural parameters as a function of the leading parameter τ. Guided by the B3LYP-D3(BJ)/def2-TZVP ab initio structures at the minimum ($\tau = \tau_0$) and at the transition state ($\tau = 0$) we have chosen the three structural relaxations expressed below (see Figure M1 for labelling), where R, $\angle$ and D denote atom distance, bond angle and dihedral angle:

$$R_{S-S} \,/\, \text{Å} = 3.9696 - 0.0919\,(\tau/\tau_0)^2 \quad \text{(M2)}$$

$$\angle(\text{C1} - \text{S1} - \text{S2}) \,/\, \text{deg} = 77.0 - 2.0\,(\tau/\tau_0)^2 - 1.0\,(\tau/\tau_0) \quad \text{(M3)}$$

$$\angle(\text{C2} - \text{S2} - \text{S1}) \,/\, \text{deg} = 77.0 - 2.0\,(\tau/\tau_0)^2 + 1.0\,(\tau/\tau_0) \quad \text{(M4)}$$

$$\angle(\text{C3} - \text{C1} - \text{S1}) \,/\, \text{deg} = 77.0 - 1.9\,(\tau/\tau_0) \quad \text{(M5)}$$

$$\angle(\text{C4} - \text{C2} - \text{S2}) \,/\, \text{deg} = 77.0 + 1.9\,(\tau/\tau_0) \quad \text{(M6)}$$

$$D(\text{C3C1} - \text{S1S2}) \,/\, \text{deg} = -71.5 + 7.8\,(\tau/\tau_0)^2 - 2.0\,(\tau/\tau_0) \quad \text{(M7)}$$

$$D(\text{C4C2} - \text{S2S1}) \,/\, \text{deg} = -71.5 + 7.8\,(\tau/\tau_0)^2 + 2.0\,(\tau/\tau_0) \quad \text{(M8)}$$

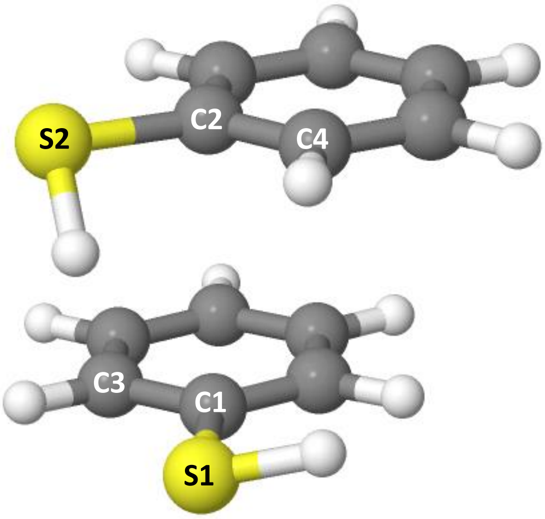

Figure M1. Definition of structural parameters used in Meyer's flexible model for the calculation of the potential barrier of equation M1.

With these structural conditions we found that B_2 = 250.3 cm^{-1} leads to a torsional splitting of ΔE_{10} = 8.88 MHz, i.e., it reproduces the experimental value. In the flexible model calculations, the τ coordinate has been considered in the ±110° range and solved into 79 mesh points.

5. *Torsional effective Hamiltonian*

The torsional potential and tunneling splitting in the thiophenol dimer II were also predicted computationally with the following procedure. Initially, geometry optimizations for two equivalent minima were performed using the DFT method PBEh-3c,[15] followed by harmonic frequency calculations. The minimal energy path (MEP) between these minima was then computed using the nudged elastic band (NEB) algorithm[33] using nine intermediate images. The obtained MEP was used as an initial guess for latter free-end NEB calculations at gCP-B3LYP-D3BJ/def2-TZVP. From the reaction path we have calculated the parameters of the effective Hamiltonian[34,35]

$$\hat{H} = \frac{1}{2}\hat{p}G(\xi)\hat{p} + V(\xi) + ZPVE(\xi) \tag{M9}$$

where ξ is the large amplitude motion (LAM) coordinate defined as ξ = (D(C$_o$'C'S'H') + D(C$_o$"C"S"H")-180°)/2, with D denoting dihedral angle between atoms given in parentheses (see Figure M2 below for atomic notation), $\hat{p} = -i\hbar\frac{\partial}{\partial\xi}$ is the momentum operator, $G(\xi)$ denotes the inverse effective mass of the motion computed as described below, while $V(\xi)$ and $ZPVE(\xi)$ stand for potential energy surface (PES), and harmonic zero-point vibrational energy of $3N-7$ vibrational modes orthogonal to LAM. The inverted effective mass $G(\xi)$ was computed as the last diagonal element of the inverted generalized tensor of intertia[30,31,34,35]

$$G = \begin{pmatrix} I_{rr} & I_{rv} \\ I_{rv}^T & I_{vv} \end{pmatrix}^{-1} \tag{M10}$$

where $I_{rr} = \sum_k m_k\,(diag(r_k \cdot r_k) - r_k \otimes r_k)$ is the 3×3 tensor of inertia, $I_{rv} = \sum_k m_k\left[r_k \times \partial_\xi r_k\right]$ is the 3D vector of rotation-LAM interaction, $I_{vv} = \sum_k m_k\left(\partial_\xi r_k \cdot \partial_\xi r_k\right)$, with k enumerating all the nuclei, m and **r** being atomic masses and Cartesian coordinates, and ·, ×, and ⊗ denoting scalar, vector, and outer products.

The parameters of Hamiltonian given by equation M9 were computed using the following algorithm.

- All the MEP structures were oriented to yield least mass-weighted deviation of the coordinates from the structure best approximating transition state (with smallest $|\xi|$). Hessians were transformed accordingly.

- The Cartesian coordinates of atoms along LAM coordinate were approximated with splines, this approximation was used to obtain derivatives $\partial_\xi r_k$.
- Seven degrees of freedom (transnational, rotational, and LAM) were removed from the Hessians, the resulting harmonic vibrational problem was solved yielding effective ZPVE correction. LAM direction was given by $\partial_\xi r_k$vectors.
- The effective structures outside the minima in the direction opposite to transitional state (the walls of the potential) were extrapolated from the coordinates of the minima via linear Taylor expansion as$r_k(\xi) \approx r_k(\xi_{eq}) + \partial_\xi r_k(\xi_{eq}) \cdot (\xi - \xi_{eq})$ with index "eq" denoting the minimal energy (equilibrium) structure. Electronic energies of these structures were estimated from the harmonic potential of the equilibrium structure, ZPVE of these structures was the ZPVE of the equilibrium structure.
- Effective masses $\mu(\xi) = G^{-1}(\xi)$ were computed from MEP and extrapolated structures by the formulas given above.
- All the resulting Hamltonian parameters were symmetrized with respect to ξ=0, and then extrapolated using cubic splines. The resulting curves are given in Figures S5 and S6.
- The effective 1D Schrödinger equation was solved using the sinc-DVR method.[36] The resulting tunneling splittings and potential barriers are given in Table S9.

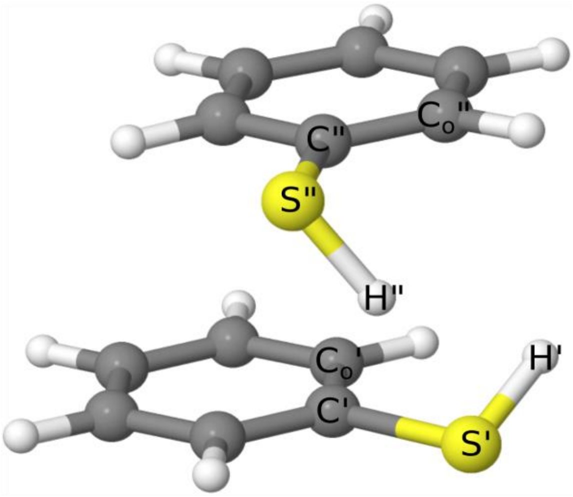

Figure M2. Dihedral angles used for definition of reaction coordinate in the effective Hamiltonian of Equation M9.

All the data needed to reproduce the results (Orca calculation results, Hamiltonian parameters and scripts) can be downloaded (zip archive) from DESY Sync&Share.[i] The software itself can also be obtained directly from the DESY repository.[ii]

[i]Available from: https://syncandshare.desy.de/index.php/s/RpYbew34JbbwdGS
[ii]Tikhonov, D.; MOLINC, **2020**, available from: https://stash.desy.de/projects/MOLINC

REFERENCES

(1) Brown, G. G.; Dian, B. C.; Douglass, K. O.; Geyer, S. M.; Shipman, S. T.; Pate, B. H. A Broadband Fourier Transform Microwave Spectrometer Based on Chirped Pulse Excitation. *Rev. Sci. Instrum.* **2008**, *79* (5), 1–13. https://doi.org/10.1063/1.2919120.

(2) Shipman, S. T.; Pate, B. H. New Techniques in Microwave Spectroscopy. In *Handbook of High-resolution Spectroscopy*; Merkt, F., Quack, M., Eds.; Major Reference Works; John Wiley & Sons, Ltd: New York, 2011; pp 801–828. https://doi.org/10.1002/9780470749593.hrs036.

(3) Grabow, J.-U. Fourier Transform Microwave Spectroscopy Measurement and Instrumentation. In *Handbook of High-resolution Spectroscopy*; Merkt, F., Quack, M., Eds.; John Wiley & Sons, Ltd: New York, 2011; pp 723–799. https://doi.org/10.1002/9780470749593.hrs037.

(4) Lesarri, A.; Pinacho, R.; Enríquez, L.; Rubio, J. E.; Jaraíz, M.; Abad, J. L.; Gigosos, M. A. Rotational Spectra of Tetracyclic Quinolizidine Alkaloids: Does a Water Molecule Flip Sparteine? *Phys. Chem. Chem. Phys.* **2017**, *19* (27), 17553–17559. https://doi.org/10.1039/c7cp01432e.

(5) Schmitz, D.; Alvin Shubert, V.; Betz, T.; Schnell, M. Multi-Resonance Effects within a Single Chirp in Broadband Rotational Spectroscopy: The Rapid Adiabatic Passage Regime for Benzonitrile. *J. Mol. Spectrosc.* **2012**, *280* (1), 77–84. https://doi.org/10.1016/j.jms.2012.08.001.

(6) Pérez, C.; Krin, A.; Steber, A. L.; López, J. C.; Kisiel, Z.; Schnell, M. Wetting Camphor: Multi-Isotopic Substitution Identifies the Complementary Roles of Hydrogen Bonding and Dispersive Forces. *J. Phys. Chem. Lett.* **2016**, *7* (1), 154–160. https://doi.org/10.1021/acs.jpclett.5b02541.

(7) Pérez, C.; Lobsiger, S.; Seifert, N. A.; Zaleski, D. P.; Temelso, B.; Shields, G. C.; Kisiel, Z.; Pate, B. H. Broadband Fourier Transform Rotational Spectroscopy for Structure Determination: The Water Heptamer. *Chem. Phys. Lett.* **2013**, *571*, 1–15. https://doi.org/10.1016/j.cplett.2013.04.014.

(8) Seifert, N. A.; Steber, A. L.; Neill, J. L.; Pérez, C.; Zaleski, D. P.; Pate, B. H.; Lesarri, A. The Interplay of Hydrogen Bonding and Dispersion in Phenol Dimer and Trimer: Structures from Broadband Rotational Spectroscopy. *Phys. Chem. Chem. Phys.* **2013**, *15* (27), 11468–11477. https://doi.org/10.1039/c3cp51725j.

(9) Halgren, T. A. Merck Molecular Force Field. II. MMFF94 van Der Waals and Electrostatic Parameters for Intermolecular Interactions. *J. Comput. Chem.* **1996**, *17* (5–6), 520–552. https://doi.org/10.1002/(SICI)1096-987X(199604)17:5/6<520::AID-JCC2>3.0.CO;2-W.

(10) MacroModel, Schrödinger Release 2020-1: Schrödinger, LLC: New York, NY 2020.

(11) Kohn, W.; Sham, L. J. Self-Consistent Equations Including Exchange and Correlation Effects. *Phys. Rev.* **1965**, *140* (4A), A1133–A1138. https://doi.org/10.1103/PhysRev.140.A1133.

(12) Becke, A. D. Density-Functional Thermochemistry. III. The Role of Exact Exchange. *J. Chem. Phys.* **1993**, *98* (7), 5648–5652. https://doi.org/10.1063/1.464913.

(13) Chai, J. Da; Head-Gordon, M. Long-Range Corrected Hybrid Density Functionals with Damped Atom-Atom Dispersion Corrections. *Phys. Chem. Chem. Phys.* **2008**, *10* (44), 6615–6620. https://doi.org/10.1039/b810189b.

(14) Grimme, S.; Neese, F. Double-Hybrid Density Functional Theory for Excited Electronic States of Molecules. *J. Chem. Phys.* **2007**, *127* (15), 1–18. https://doi.org/10.1063/1.2772854.

(15) Grimme, S.; Brandenburg, J. G.; Bannwarth, C.; Hansen, A. Consistent Structures and Interactions by Density Functional Theory with Small Atomic Orbital Basis Sets. *J. Chem. Phys.*

2015, *143* (5), 054107. https://doi.org/10.1063/1.4927476.
(16) Grimme, S.; Antony, J.; Ehrlich, S.; Krieg, H. A Consistent and Accurate Ab Initio Parametrization of Density Functional Dispersion Correction (DFT-D) for the 94 Elements H-Pu. *J. Chem. Phys.* **2010**, *132* (15), 1–19. https://doi.org/10.1063/1.3382344.
(17) Johnson, E. R.; Becke, A. D. A Post-Hartree-Fock Model of Intermolecular Interactions: Inclusion of Higher-Order Corrections. *J. Chem. Phys.* **2006**, *124* (17), 174104. https://doi.org/10.1063/1.2190220.
(18) Grimme, S.; Ehrlich, S.; Goerigk, L. Effect of the Damping Function in Dispersion Corrected Density Functional Theory. *J. Comput. Chem.* **2011**, *32* (7), 1456–1465. https://doi.org/10.1002/jcc.21759.
(19) Weigend, F.; Ahlrichs, R. Balanced Basis Sets of Split Valence, Triple Zeta Valence and Quadruple Zeta Valence Quality for H to Rn: Design and Assessment of Accuracy. *Phys. Chem. Chem. Phys.* **2005**, *7* (18), 3297. https://doi.org/10.1039/b508541a.
(20) Wilson, A. K.; van Mourik, T.; Dunning, T. H. Gaussian Basis Sets for Use in Correlated Molecular Calculations. VI. Sextuple Zeta Correlation Consistent Basis Sets for Boron through Neon. *J. Mol. Struct. THEOCHEM* **1996**, *388*, 339–349. https://doi.org/10.1016/S0166-1280(96)80048-0.
(21) Frisch, M. J.; Trucks, G. W.; Schlegel, H. B.; Scuseria, G. E.; Robb, M. A.; Cheeseman, J. R.; Scalmani, G.; Barone, V.; Petersson, G. A.; Nakatsuji, H.; Li, X.; Caricato, M.; Marenich, A. V.; Bloino, J.; Janesko, B. G.; Gomperts, R.; Mennucci, B.; Hratchian, H. P.; Ortiz, J. V.; Izmaylov, A. F.; Sonnenberg, J. L.; Williams; Ding, F.; Lipparini, F.; Egidi, F.; Goings, J.; Peng, B.; Petrone, A.; Henderson, T.; Ranasinghe, D.; Zakrzewski, V. G.; Gao, J.; Rega, N.; Zheng, G.; Liang, W.; Hada, M.; Ehara, M.; Toyota, K.; Fukuda, R.; Hasegawa, J.; Ishida, M.; Nakajima, T.; Honda, Y.; Kitao, O.; Nakai, H.; Vreven, T.; Throssell, K.; Montgomery Jr., J. A.; Peralta, J. E.; Ogliaro, F.; Bearpark, M. J.; Heyd, J. J.; Brothers, E. N.; Kudin, K. N.; Staroverov, V. N.; Keith, T. A.; Kobayashi, R.; Normand, J.; Raghavachari, K.; Rendell, A. P.; Burant, J. C.; Iyengar, S. S.; Tomasi, J.; Cossi, M.; Millam, J. M.; Klene, M.; Adamo, C.; Cammi, R.; Ochterski, J. W.; Martin, R. L.; Morokuma, K.; Farkas, O.; Foresman, J. B.; Fox, D. J. Gaussian 16, Rev. C.01. Gaussian, Inc: Wallingford CT 2016.
(22) Neese, F. Software Update: The ORCA Program System, Version 4.0. *WIREs Comput. Mol. Sci.* **2018**, *8* (1). https://doi.org/10.1002/wcms.1327.
(23) Johnson, E. R.; Keinan, S.; Mori-Sánchez, P.; Contreras-García, J.; Cohen, A. J.; Yang, W. Revealing Noncovalent Interactions. *J. Am. Chem. Soc.* **2010**, *132* (18), 6498–6506. https://doi.org/10.1021/ja100936w.
(24) Contreras-García, J.; Johnson, E. R.; Keinan, S.; Chaudret, R.; Piquemal, J. P.; Beratan, D. N.; Yang, W. NCIPLOT: A Program for Plotting Noncovalent Interaction Regions. *J. Chem. Theory Comput.* **2011**, *7* (3), 625–632. https://doi.org/10.1021/ct100641a.
(25) Scheiner, S. *Hydrogen Bonding: A Theoretical Perspective*; Press, O. U., Ed.; Oxford, 1997.
(26) Jeziorski, B.; Moszynski, R.; Szalewicz, K. Perturbation Theory Approach to Intermolecular Potential Energy Surfaces of van Der Waals Complexes. *Chem. Rev.* **1994**, *94* (7), 1887–1930. https://doi.org/10.1021/cr00031a008.
(27) Parrish, R. M.; Burns, L. A.; Smith, D. G. A.; Simmonett, A. C.; DePrince, A. E.; Hohenstein, E. G.; Bozkaya, U.; Sokolov, A. Y.; Di Remigio, R.; Richard, R. M.; Gonthier, J. F.; James, A. M.; McAlexander, H. R.; Kumar, A.; Saitow, M.; Wang, X.; Pritchard, B. P.; Verma, P.; Schaefer, H. F.; Patkowski, K.; King, R. A.; Valeev, E. F.; Evangelista, F. A.; Turney, J. M.; Crawford, T. D.; Sherrill, C. D. Psi4 1.1: An Open-Source Electronic Structure Program Emphasizing Automation, Advanced Libraries, and Interoperability. *J. Chem. Theory Comput.* **2017**, *13* (7),

3185–3197. https://doi.org/10.1021/acs.jctc.7b00174.
(28) Hohenstein, E. G.; Sherrill, C. D. Density Fitting of Intramonomer Correlation Effects in Symmetry-Adapted Perturbation Theory. *J. Chem. Phys.* **2010**, *133* (1), 1–12. https://doi.org/10.1063/1.3451077.
(29) Meyer, R. Flexible Models for Intramolecular Motion, a Versatile Treatment and Its Application to Glyoxal. *J. Mol. Spectrosc.* **1979**, *76* (1–3), 266–300. https://doi.org/10.1016/0022-2852(79)90230-3.
(30) Sørensen, G. O. A New Approach to the Hamiltonian of Nonrigid Molecules. In *Topics in Current Chemistry 82: Large Amplitude Motion in Molecules II*; Springer-Verlag: Berlin/Heidelberg, 1979; pp 97–175. https://doi.org/10.1007/BFb0048009.
(31) Bauder, A.; Mathier, E.; Meyer, R.; Ribeaud, M.; Günthard, H. H. Theory of Rotation and Torsion Spectra for a Semi-Rigid Model of Molecules with an Internal Rotor of C 2 v Symmetry. *Mol. Phys.* **1968**, *15* (6), 597–614. https://doi.org/10.1080/00268976800101501.
(32) Caminati, W. Microwave Spectroscopy of Large Molecules and Molecular Complexes. In *Handbook of High-resolution Spectroscopy*; Merkt, F., Quack, M., Eds.; John Wiley & Sons, Ltd: New York, NY, 2011. https://doi.org/https://doi.org/10.1002/9780470749593.hrs035.
(33) Mills, G.; Jónsson, H.; Schenter, G. K. Reversible Work Transition State Theory: Application to Dissociative Adsorption of Hydrogen. *Surf. Sci.* **1995**, *324* (2–3), 305–337. https://doi.org/10.1016/0039-6028(94)00731-4.
(34) Kochikov, I. V.; Tarasov, Y. I.; Vogt, N.; Spiridonov, V. P. Large-Amplitude Motion in 1,4-Cyclohexadiene and 1,4-Dioxin: Theoretical Background for Joint Treatment of Spectroscopic, Electron Diffraction and Ab Initio Data. *J. Mol. Struct.* **2002**, *607* (2–3), 163–174. https://doi.org/10.1016/S0022-2860(01)00914-0.
(35) Kochikov, I. V; Tarasov, Y. I. Equilibrium Structure and Internal Rotation in B2F4 from Electron Diffraction and Spectroscopic Data and Quantum Chemical Calculations. *Struct. Chem.* **2003**, *14* (2), 227–238. https://doi.org/10.1023/A:1022102918764.
(36) Colbert, D. T.; Miller, W. H. A Novel Discrete Variable Representation for Quantum Mechanical Reactive Scattering via the S -matrix Kohn Method. *J. Chem. Phys.* **1992**, *96* (3), 1982–1991. https://doi.org/10.1063/1.462100.

Figure S1. A section of the microwave spectrum of thiophenol and its aggregates, illustrating the larger (ca. 17 MHz) tunnelling splittings in the μ_c inter-state (v = 1 $\leftrightarrow$ 0) torsional-rotational transitions of the thiophenol dimer global minimum PD2-cis. The intra-state (v = 0 $\leftrightarrow$ 0, 1 $\leftrightarrow$ 1) μ_b transitions display smaller splittings of ca. 1 MHz.

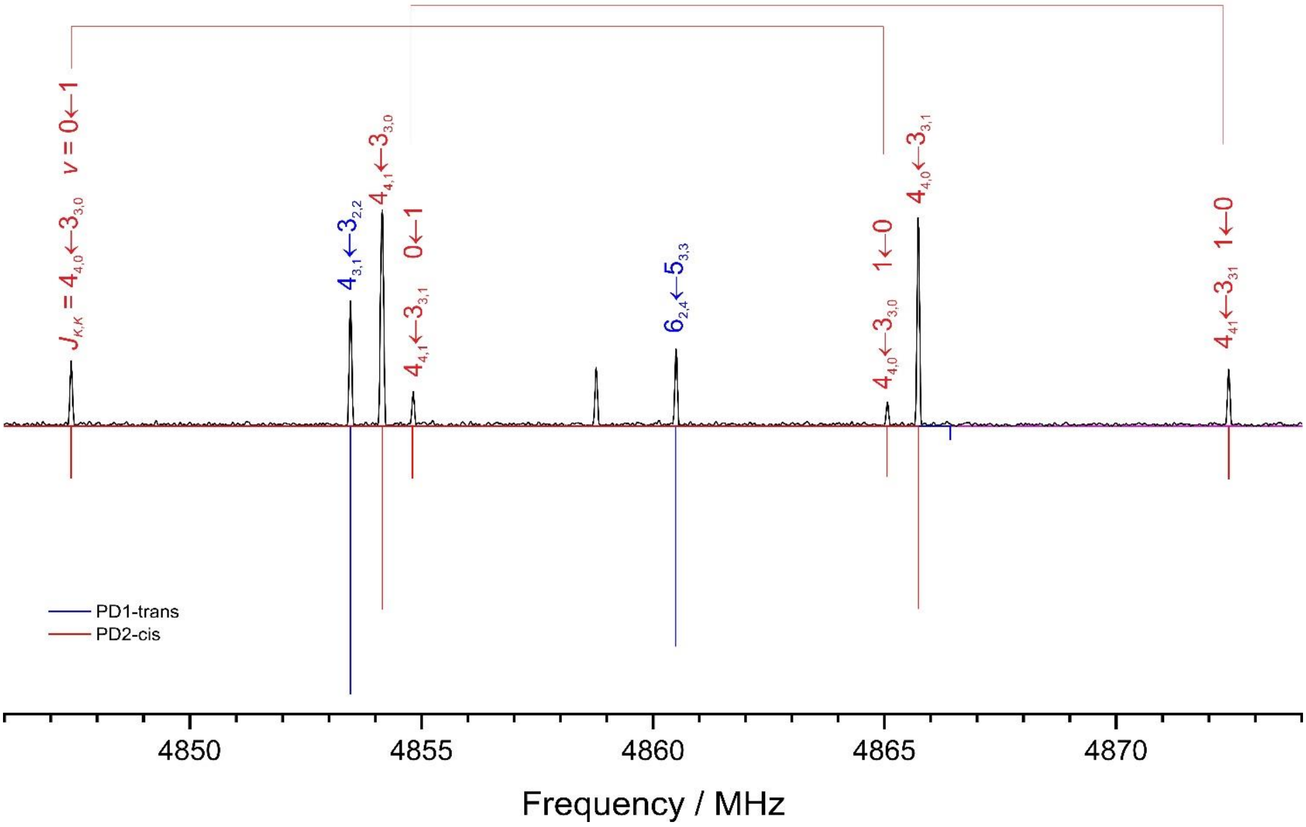

Figure S2. 3D Figures of the parallel-displaced-1 (PD1 *cis* and *trans*) isomers of the thiophenol dimer.

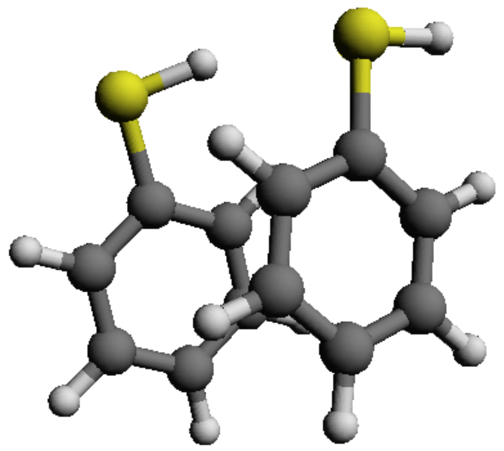

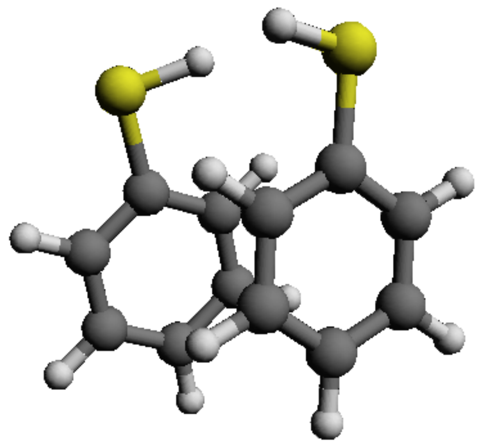

Figure S3. 3D Figures of the parallel-displaced-2 (PD2, *cis* and *trans*) isomers of the thiophenol dimer.

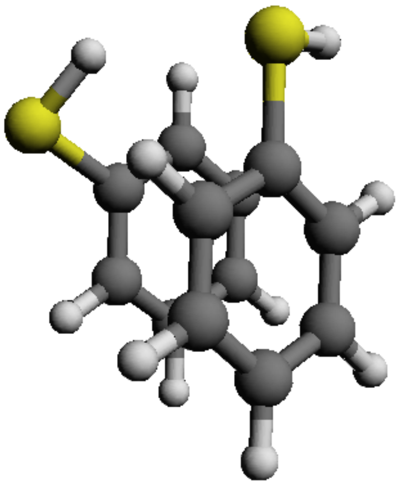

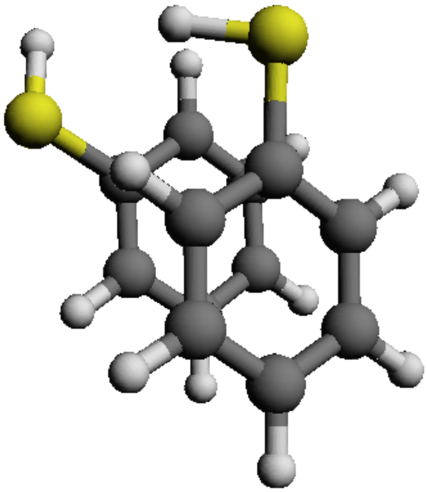

Figure S4. Principal inertial axes system for the isomer PD2-cis of the thiophenol dimer. The thiol internal rotation would contribute to the inversion of the μ_c electric dipole moment component.

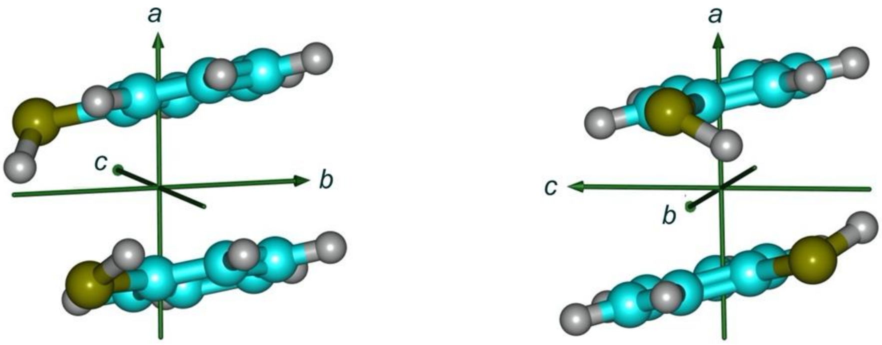

Figure S5. Computed PES ($V(\xi)$, left side of the figure) and effective PES ($V(\xi) + ZPVE(\xi)$, right side of the figure) for the thiophenol dimer, according to the monodimensional torsional Hamiltonian of equation M9.

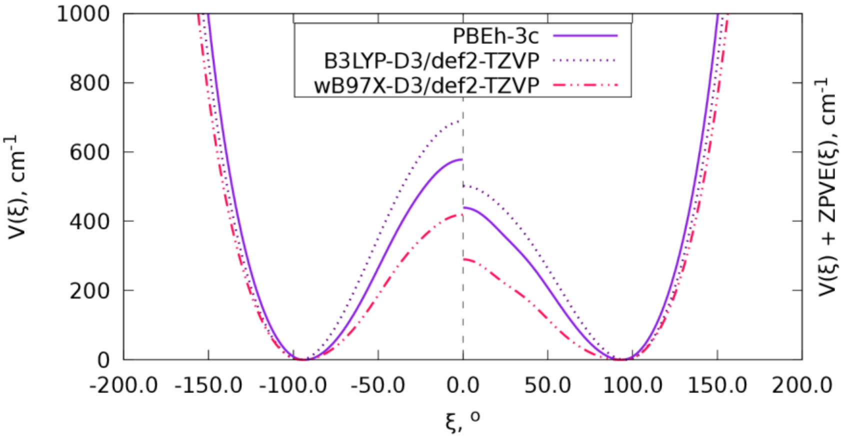

Figure S6. Computed PES effective mass $\mu(\xi) = G^{-1}(\xi)$ of the LAM in the thiophenol dimer along the reaction coordinate, according to the monodimensional torsional Hamiltonian of equation M9.

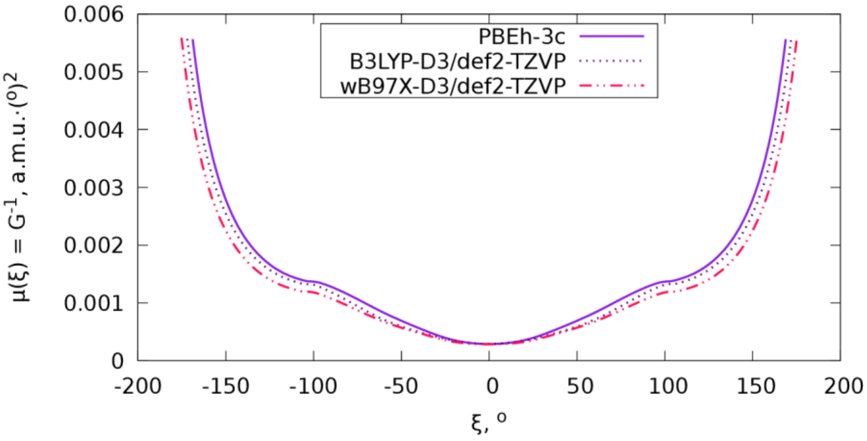

Figure S7. A 20 MHz section of the rotational spectrum of the thiophenol dimer, showing a comparison of the spectral intensities when using Ne (upper trace) or Ar (lower trace) as carrier gas in the jet expansion (see also Figures 2 and 3). The disappearance of isomer PD1 with Ar confirms PD2 as global minimum.

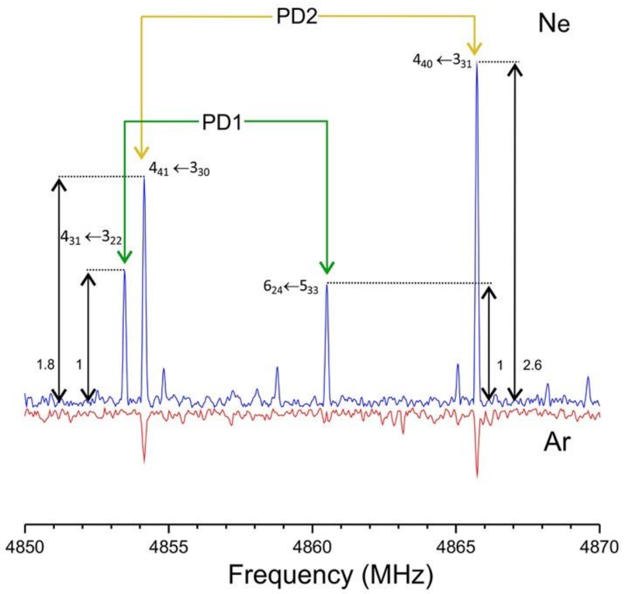

Figure S8. 3D Figure of the symmetric rotor isomer (UUU, see Table S3) of the thiophenol trimer.

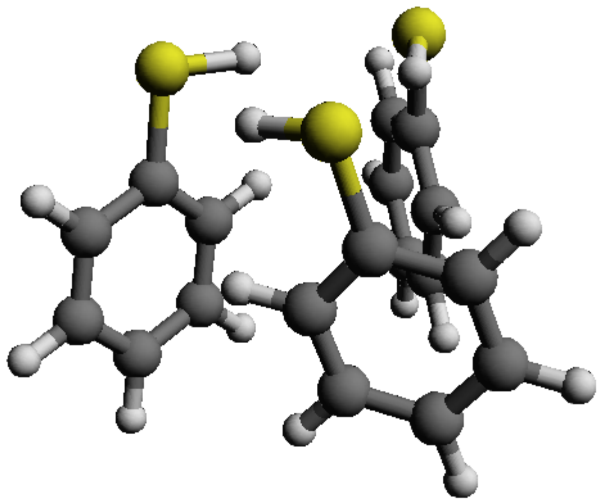

Figure S9. NCIPlot mapping the non-convalent interactions for the second isomer of the thiophenol dimer PD1-trans, showing a primary hydrogen bond S-H···S (blue) and diffuse interplanar attractive interactions (green). Repulsive ring critical points are indicated in red.

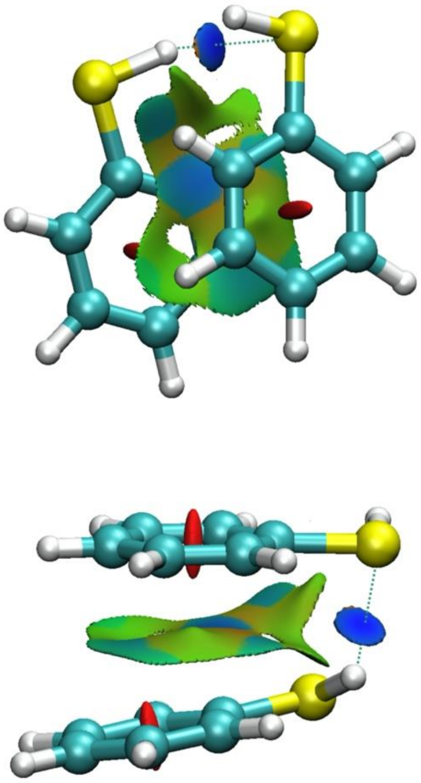

Table S1. Conformational search for the thiophenol dimer, according to B3LYP-D3(BJ)/def2-TZVP.

Isomer	1	2	3	4
	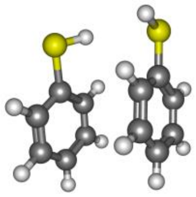	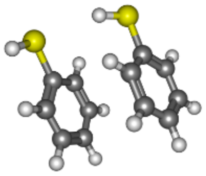	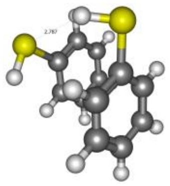	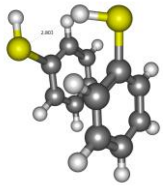
A / MHz [a]	690.7	688.3	630.0	633.9
B / MHz	498.6	499.4	527.3	525.9
C / MHz	346.5	349.6	433.6	434.5
Δ_J / kHz	0.337	0.287	0.085	0.122
Δ_{JK} / kHz	-0.456	-0.299	0.243	0.065
Δ_K / kHz	0.165	0.062	-0.268	-0.121
δ_J / kHz	0.033	0.018	-0.022	-0.021
δ_K / kHz	0.107	0.193	0.435	0.259
$\lvert\mu_a\rvert$ / D	0.1	0.6	0.0	0.7
$\lvert\mu_b\rvert$ / D	1.5	2.0	1.6	1.1
$\lvert\mu_c\rvert$ / D	0.3	0.9	1.1	0.2
ΔE / kJ mol^{-1} [b]	0.6	1.4	0.0	0.4
$\Delta G_{298\ K}$ / kJ mol^{-1}	0.00	0.77	2.18	2.46
ΔE_c / kJ mol^{-1}	-29.62	-28.83	-31.34	-31.17
r(S-H···S) / Å [c]	2.838	2.807	2.787	2.803
∠ (S-H···S) / deg	138.8	141.6	136.7	135.4

[a]Rotational constants (A, B, C), Watson's A-reduction centrifugal distortion constants (Δ_J, Δ_{JK}, δ_K, δ_J, δ_K) and electric dipole moments (μ_α, α = a, b, c). [b]Relative energies corrected with the zero-point energy (ΔE), Gibbs energy (ΔG, 100 K and 298K, 1 atm) and complexation energy (ΔE_c). [c]Structural parameters for the S-H···S hydrogen bond. All values calculated with B3LYP-D3(BJ)/def2-TVZP.

Table S1. Continued.

Isomer	5	6	7	8
	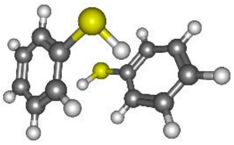	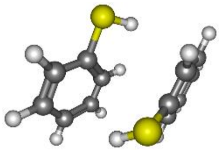	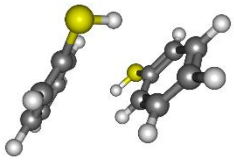	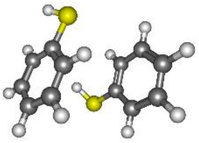
A / MHz [a]	825.4	736.6	710.7	700.5
B / MHz	412.8	430.2	448.5	452.5
C / MHz	356.1	375.6	353.0	363.4
Δ_J / kHz	0.083	0.130	0.164	0.205
Δ_{JK} / kHz	-0.277	0.147	-0.259	-0.370
Δ_K / kHz	0.590	-0.438	0.350	0.442
δ_J / kHz	0.022	0.027	0.019	0.036
δ_K / kHz	0.135	0.112	0.230	0.261
$\lvert\mu_a\rvert$ / D	0.0	0.5	0.3	0.9
$\lvert\mu_b\rvert$ / D	0.0	0.5	0.3	0.4
$\lvert\mu_c\rvert$ / D	1.2	1.6	1.2	0.6
ΔE / kJ mol^{-1} [b]	2.1	2.9	3.1	4.7
$\Delta G_{298\ K}$ / kJ mol^{-1}	3.68	1.82	0.77	1.75
ΔE_c / kJ mol^{-1}	-30.79	-29.08	-28.58	-27.53

[a]Rotational constants (A, B, C), Watson's A-reduction centrifugal distortion constants (Δ_J, Δ_{JK}, δ_K, δ_J, δ_K) and electric dipole moments (μ_α, $\alpha = a, b, c$). [b]Relative energies corrected with the zero-point energy (ΔE), Gibbs energy (ΔG, 100 K and 298K, 1 atm) and complexation energy (ΔE_c). All values calculated with B3LYP-D3(BJ)/def2-TVZP.

Table S2. Conformational search for the thiophenol dimer, according to ωB97XD/cc-PVTZ.

Isomer	1	2	3	4
	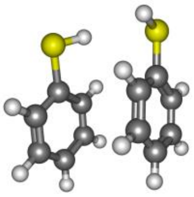	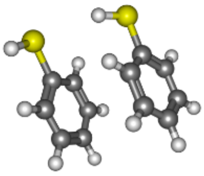	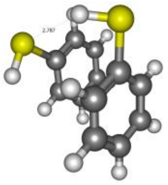	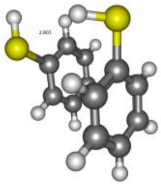
A / MHz [a]	687.0	691.5	628.0	629.4
B / MHz	495.7	488.7	518.5	522.1
C / MHz	345.1	345.8	430.9	428.5
Δ_J / kHz	0.385	0.259	0.057	0.107
Δ_{JK} / kHz	-0.626	-0.418	0.292	0.289
Δ_K / kHz	0.283	0.206	-0.282	-0.321
δ_J / kHz	0.051	0.020	-0.016	-0.025
δ_K / kHz	0.009	-0.027	0.419	0.521
$\lvert\mu_a\rvert$ / D	0.1	0.5	0.1	-0.8
$\lvert\mu_b\rvert$ / D	1.7	2.3	1.9	1.3
$\lvert\mu_c\rvert$ / D	0.4	0.9	1.1	-0.2
ΔE / kJ mol^{-1} [b]	0.00	1.88	0.04	1.38
$\Delta G_{298\ K}$ / kJ mol^{-1}	0.00	4.03	3.52	4.89
ΔE_c / kJ mol^{-1}	-26.48	-25.61	-27.87	-26.99
r(S-H···S) / Å [c]	2.933	2.889	2.870	2.886
∠ (S-H···S) / deg	139.1	146.0	138.5	135.7

[a]Rotational constants (A, B, C), Watson's A-reduction centrifugal distortion constants (Δ_J, Δ_{JK}, δ_K, δ_J, δ_K) and electric dipole moments (μ_α, α = a, b, c). [b]Relative energies corrected with the zero-point energy (ΔE), Gibbs energy (ΔG, 100 K and 298K, 1 atm) and complexation energy (ΔE_c). [c]Structural parameters for the S-H···S hydrogen bond. All values calculated with ωB97XD/cc-PVTZ.

Table S3. Conformational search for the thiophenol trimer, according to B3LYP-D3(BJ)/def2-TZVP.

Isomer	1<br>UUU	2<br>UUD	3	4
	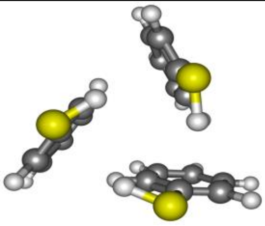	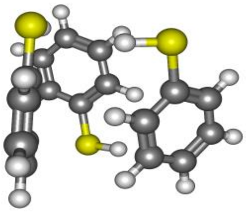	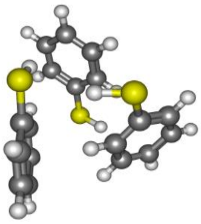	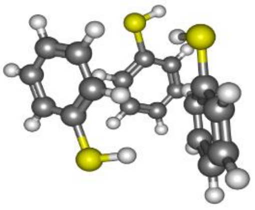
A / MHz [a]	234.3	241.8	235.9	250.4
B / MHz	234.3	232.9	235.1	228.9
C / MHz	202.2	195.9	198.9	192.1
Δ_J / kHz	0.012	0.012	0.015	0.039
Δ_{JK} / kHz	0.105	0.020	0.065	-0.023
Δ_K / kHz	-0.111	-0.024	-0.068	-0.005
δ_J / kHz	0.000	0.002	-0.001	0.009
δ_K / kHz	-0.358	0.032	0.231	-0.068
$\|\mu_a\|$ / D	0.0	0.4	0.2	0.5
$\|\mu_b\|$ / D	0.0	0.3	0.1	1.2
$\|\mu_c\|$ / D	3.0	0.9	1.0	1.0
ΔE / kJ mol^{-1} [b]	0.0	0.24	0.97	3.69
$\Delta G_{298\ K}$ /kJ mol^{-1}	0.0	2.14	6.93	2.43
ΔE_c / kJ mol^{-1}	-78.07	-77.07	-76.27	-72.97

[a]Rotational constants (*A*, *B*, *C*), Watson's A-reduction centrifugal distortion constants (Δ_J, Δ_{JK}, δ_K, δ_J, δ_K) and electric dipole moments (μ_α, α = *a*, *b*, *c*). [b]Relative energies corrected with the zero-point energy (ΔE), Gibbs energy (ΔG, 100 K and 298K, 1 atm) and complexation energy (ΔE_c). All values calculated with B3LYP-D3(BJ)/def2-TVZP.

Table S3. Continued.

Isomer	5	6	7	8
	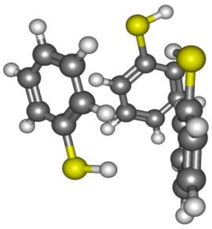	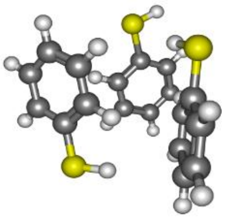	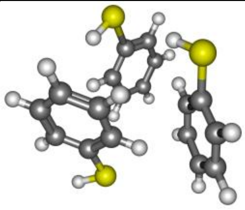	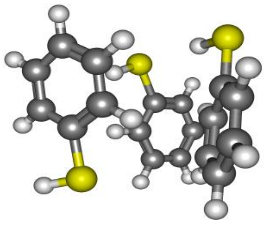
A / MHz [a]	249.6	244.8	246.2	247.1
B / MHz	229.5	233.3	230.5	217.3
C / MHz	192.3	193.7	195.9	193.3
Δ_J / kHz	0.055	0.012	0.012	0.126
Δ_{JK} / kHz	-0.032	0.063	0.000	-0.298
Δ_K / kHz	-0.012	-0.067	-0.005	0.253
δ_J / kHz	0.011	0.001	0.002	-0.019
δ_K / kHz	-0.098	0.164	-0.003	-0.278
$\lvert\mu_a\rvert$ / D	0.5	0.8	1.2	0.3
$\lvert\mu_b\rvert$ / D	1.2	0.7	1.9	1.9
$\lvert\mu_c\rvert$ / D	1.0	1.1	1.0	1.2
ΔE / kJ mol^{-1} [b]	3.83	4.10	4.54	5.15
$\Delta G_{298\,K}$ /kJ mol^{-1}	2.45	4.67	6.97	8.39
ΔE_c / kJ mol^{-1}	-72.97	-73.22	-73.01	-71.55

[a]Rotational constants (A, B, C), Watson's A-reduction centrifugal distortion constants (Δ_J, Δ_{JK}, δ_K, δ_J, δ_K) and electric dipole moments (μ_α, α = a, b, c). [b]Relative energies corrected with the zero-point energy (ΔE), Gibbs energy (ΔG, 100 K and 298K, 1 atm) and complexation energy (ΔE_c). All values calculated with B3LYP-D3(BJ)/def2-TVZP.

Table S4. Predictions for the most stable species of the thiophenol trimer according to ωB97XD/cc-PVTZ.

Isomer	1<br>UUU	2<br>UUD
	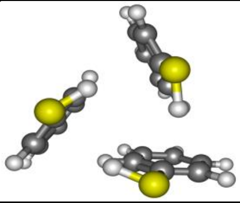	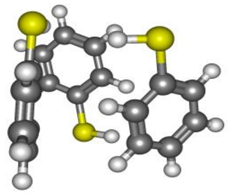
A / MHz[a]	236.1	243.6
B / MHz	236.0	230.1
C / MHz	200.3	191.7
Δ_J / kHz	0.009	0.011
Δ_{JK} / kHz	0.019	-0.004
Δ_K / kHz	-0.022	0.000
δ_J / kHz	0.000	0.001
δ_K / kHz	-0.180	-0.006
$\|\mu_a\|$ / D	0.0	0.7
$\|\mu_b\|$ / D	0.0	0.2
$\|\mu_c\|$ / D	3.3	0.9
ΔE / kJ mol^{-1} [b]	0	-0.74
$\Delta G_{298\ K}$ / kJ mol^{-1}	0.00	-1.16
ΔE_c / kJ mol^{-1}	-70.79	-71.00

[a]Rotational constants (*A*, *B*, *C*), Watson's A-reduction centrifugal distortion constants (Δ_J, Δ_{JK}, δ_K, δ_J, δ_K) and electric dipole moments (μ_α, α = *a*, *b*, *c*). [b]Relative energies corrected with the zero-point energy (ΔE), Gibbs energy (ΔG, 100 K and 298K, 1 atm) and complexation energy (ΔE_c). All values calculated with ωB97XD/cc-PVTZ.

Table S5. Rotational parameters for the thiophenol monomer and DFT predictions.

	Experiment[b]		Theory[d]		
	0^{+} [c]	0^{-}	B3LYP	B2PLYP	ωB97XD
A / MHz[a]	5588.887(2)	-1.4604(7)	5640.29	5631.99	5666.96
B / MHz	1577.8588(6)	0.00827(3)	1581.14	1581.68	1585.76
C / MHz	1231.0865(4)	0.00060(23)	1234.95	1234.88	1239.05
Δ_J / kHz	0.0630(12)		0.061	0.061	0.060
Δ_{JK} / kHz	0.282(8)		0.269	0.272	0.268
Δ_K / kHz	0.76(10)		0.803	0.807	0.805
δ_J / kHz	-0.015548(17)		0.015	0.015	0.015
δ_K / kHz	-0.305(8)		0.301	0.302	0.299
ΔE_0 / MHz	821.999(15)				
F_{ab} / MHz	19.31(5)				
$\|\mu_a\|$ / D			0.7	0.8	0.9
$\|\mu_b\|$ / D			0.8	0.8	0.8
$\|\mu_c\|$ / D			0.0	0.0	0.0

[a]Rotational constants (A, B, C), Watson's A-reduction centrifugal distortion constants (Δ_J, Δ_{JK}, δ_K, δ_J, δ_K), energy difference between torsional substates (ΔE_0), Coriolis coupling parameter (F_{ab}) for operator (P_aP_b+P_bP_a) and electric dipole moments (μ_α, α = a, b, c). [b]N W. Larsen, L. Schulz, *J. Mol. Struct*. 920 (2009), 30-39. [c]Torsional substates 0^+ and 0^-, indicating A, B, C and ΔA, ΔB and ΔC for 0^+ and 0^-, respectively. [d]Calculations using B3LYP-D3(BJ)/def2-TZVP, B2PLYP-D3(BJ)/def2-TZVP and ωB97XD/cc-PVTZ, respectively.

Table S6. Observed rotational transitions for isomer PD1-trans of the thiophenol dimer, with experimental frequencies (Freq) and residuals (o.-c.) according to the Fit of Table 1.

J'	*K-1'*	*K+1'*	*J'*	*K-1'*	*K+1'*	Freq / MHz	o.-c. / MHz
6	3	4	6	2	5	2025.3504	-0.0049
5	2	4	5	1	5	2030.3324	0.0015
6	5	2	6	4	3	2040.6251	0.0020
5	5	1	5	4	2	2050.5385	0.0125
7	4	4	7	3	5	2076.3770	-0.0036
7	5	3	7	4	4	2084.6215	0.0009
8	3	5	8	2	6	2152.9099	0.0010
8	6	2	8	5	3	2177.2853	0.0000
3	1	2	2	2	1	2202.3384	0.0055
3	0	3	2	1	2	2208.4045	-0.0019
8	5	4	8	4	5	2217.1030	-0.0064
3	1	3	2	0	2	2309.9402	0.0009
2	2	1	1	1	0	2326.6716	0.0072
7	2	5	7	1	6	2352.4735	-0.0069
7	6	1	7	5	2	2379.7194	-0.0050
8	4	5	8	3	6	2415.5355	-0.0057
8	6	3	8	5	4	2431.9039	0.0008
7	3	5	7	2	6	2434.0439	-0.0037
9	5	5	9	4	6	2454.5491	-0.0029
6	1	5	6	0	6	2457.3174	0.0057
7	6	2	7	5	3	2457.5679	0.0091
9	6	4	9	5	5	2462.5479	0.0069
6	2	5	6	1	6	2473.5590	0.0070
6	6	0	6	5	1	2487.1916	0.0066
10	7	3	10	6	4	2491.9559	0.0022
6	6	1	6	5	2	2502.4853	0.0123
2	2	0	1	1	1	2561.4730	0.0089
10	6	5	10	5	6	2584.7657	0.0142
9	3	6	9	2	7	2711.7481	-0.0010
4	2	2	3	3	1	2735.2024	-0.0079
9	7	2	9	6	3	2739.0735	-0.0065
10	5	6	10	4	7	2788.6484	0.0035
9	4	6	9	3	7	2820.6076	-0.0044
9	7	3	9	6	4	2844.9156	0.0124
8	2	6	8	1	7	2848.4104	-0.0105
8	3	6	8	2	7	2875.1589	-0.0021
8	7	1	8	6	2	2882.2112	-0.0002
8	7	2	8	6	3	2907.7688	0.0032
4	0	4	3	1	3	2922.2621	-0.0011
7	1	6	7	0	7	2922.7017	0.0029
7	2	6	7	1	7	2927.0665	0.0061

4	1	4	3	0	3	2954.3581	0.0012
7	7	0	7	6	1	2962.4821	0.0074
3	2	2	2	1	1	3003.3322	0.0028
11	4	7	11	3	8	3058.3755	0.0169
11	8	3	11	7	4	3078.9274	0.0166
4	1	3	3	2	2	3141.1917	0.0002
11	5	7	11	4	8	3192.5167	0.0130
10	3	7	10	2	8	3224.3646	-0.0047
10	8	2	10	7	3	3257.9352	0.0095
10	4	7	10	3	8	3261.7879	0.0002
10	8	3	10	7	4	3294.8169	-0.0003
9	2	7	9	1	8	3321.5118	-0.0065
9	3	7	9	2	8	3329.2328	0.0088
9	8	1	9	7	2	3365.3674	-0.0101
9	8	2	9	7	3	3372.8124	-0.0116
8	1	7	8	0	8	3382.3708	0.0022
8	2	7	8	1	8	3383.4455	0.0048
8	8	0	8	7	1	3435.1968	-0.0016
8	8	1	8	7	2	3436.2091	-0.0062
12	4	8	12	3	9	3587.6594	0.0093
4	2	3	3	1	2	3602.3447	0.0039
5	0	5	4	1	4	3610.8329	-0.0019
5	1	5	4	0	4	3619.3016	-0.0007
12	5	8	12	4	9	3635.2206	0.0132
3	3	1	2	2	0	3697.4158	0.0038
11	3	8	11	2	9	3706.3794	-0.0190
11	4	8	11	3	9	3717.6840	-0.0154
10	2	8	10	1	9	3784.7932	-0.0177
10	3	8	10	2	9	3786.8297	-0.0028
3	2	1	2	1	2	3789.3816	-0.0009
3	3	0	2	2	1	3795.4554	0.0035
5	2	3	4	3	2	3839.2861	0.0013
5	1	4	4	2	3	3973.6882	0.0009
12	3	9	12	2	10	4174.3252	-0.0169
5	2	4	4	1	3	4178.5371	0.0005
11	3	9	11	2	10	4244.5907	-0.0108
6	0	6	5	1	5	4290.9833	0.0012
6	1	6	5	0	5	4292.9913	0.0015
10	2	9	10	1	10	4296.7458	0.0107
6	3	3	5	4	2	4302.0154	-0.0071
4	3	2	3	2	1	4405.8510	-0.0006
6	1	5	5	2	4	4717.9655	0.0027
11	1	10	11	0	11	4752.9223	0.0182
11	2	10	11	1	11	4752.9223	0.0073
6	2	5	5	1	4	4789.5110	0.0142
8	5	3	7	6	2	4838.8835	0.0022
4	3	1	3	2	2	4853.4682	-0.0014
6	2	4	5	3	3	4860.4993	-0.0020

7	0	7	6	1	6	4968.8144	0.0055
7	1	7	6	0	6	4969.2557	0.0038
5	3	3	4	2	2	5004.4407	0.0002
4	4	1	3	3	0	5054.2341	0.0025
4	4	0	3	3	1	5084.7297	0.0054
12	1	11	12	0	12	5208.7839	0.0130
12	2	11	12	1	12	5208.7839	0.0109
4	2	2	3	1	3	5245.1873	-0.0039
7	1	6	6	2	5	5417.9588	0.0031
7	2	6	6	1	5	5439.0072	0.0065
7	3	4	6	4	3	5499.7001	0.0083
6	3	4	5	2	3	5530.9778	0.0015
8	0	8	7	1	7	5646.0791	0.0014
8	1	8	7	0	7	5646.1712	0.0011
13	1	12	13	0	13	5664.3414	-0.0022
13	2	12	13	1	13	5664.3414	-0.0026
7	2	5	6	3	4	5745.0881	0.0073
5	4	2	4	3	1	5829.0285	0.0041
8	4	4	7	5	3	5896.4451	-0.0037
5	4	1	4	3	2	6025.1853	0.0028
7	3	5	6	2	4	6052.7719	-0.0024
8	1	7	7	2	6	6101.3741	-0.0115
8	2	7	7	1	6	6106.9184	0.0062
5	3	2	4	2	3	6141.9118	0.0008
9	0	9	8	1	8	6323.2331	0.0086
9	1	9	8	0	8	6323.2331	-0.0097
5	5	1	4	4	0	6390.4872	0.0039
5	5	0	4	4	1	6398.4259	-0.0001
6	4	3	5	3	2	6476.4860	-0.0019
8	2	6	7	3	5	6515.7645	0.0054
8	3	5	7	4	4	6592.3027	0.0154
8	3	6	7	2	5	6629.5871	-0.0056
9	1	8	8	2	7	6779.7517	0.0031
9	2	8	8	1	7	6781.0791	-0.0061
10	0	10	9	1	9	7000.3454	0.0023
10	1	10	9	0	9	7000.3454	-0.0011
7	4	4	6	3	3	7002.5960	-0.0071
6	4	2	5	3	3	7124.8360	-0.0048
9	4	5	8	5	4	7178.3666	0.0173
6	5	2	5	4	1	7215.5604	-0.0008
9	2	7	8	3	6	7226.1122	0.0064
9	3	7	8	2	6	7261.8957	0.0074
6	5	1	5	4	2	7283.2590	-0.0097
10	1	9	9	2	8	7456.8163	0.0023
10	2	9	9	1	8	7457.1175	0.0007
8	4	5	7	3	4	7460.6527	-0.0108
9	3	6	8	4	5	7522.2963	-0.0172
6	3	3	5	2	4	7664.7869	-0.0015

11	0	11	10	1	10	7677.4554	0.0060
11	1	11	10	0	10	7677.4554	0.0054
6	6	1	5	5	0	7718.0450	-0.0140
6	6	0	5	5	1	7719.9092	-0.0184
10	2	8	9	3	7	7912.3956	-0.0053
10	3	8	9	2	7	7922.4287	-0.0022
9	4	6	8	3	5	7929.5767	-0.0147
7	5	3	6	4	2	7949.4287	-0.0073

Table S7. Observed rotational transitions for isomer PD2-cis of the thiophenol dimer, with experimental frequencies (Freq) and residuals (o.-c.) according to the Fit of Table 1.

J'	K_{-1}'	K_{+1}'	*v'*	*J'*	K_{-1}'	K_{+1}'	*v''*	Freq / MHz	o.-c. / MHz
2	1	1	0	1	0	1	1	2152.3433	0.0037
2	1	1	1	1	0	1	0	2169.9913	0.0050
10	8	3	0	10	7	4	0	2228.5489	0.0350
10	8	3	1	10	7	4	1	2228.6278	-0.0723
9	2	8	0	9	1	9	0	2252.8179	-0.0178
2	2	1	1	1	1	0	1	2303.0594	0.0033
2	2	1	0	1	1	0	0	2303.0819	-0.0197
2	2	0	0	1	1	0	1	2329.1686	-0.0093
2	2	0	1	1	1	0	0	2346.9020	0.0074
2	2	1	0	1	1	1	1	2382.8105	-0.0022
2	2	1	1	1	1	1	0	2400.4557	-0.0052
2	2	0	0	1	1	1	0	2426.5909	0.0082
2	2	0	1	1	1	1	1	2426.5909	-0.0149
3	1	2	1	2	2	1	1	2561.9787	0.0033
3	1	2	0	2	2	1	0	2562.0764	0.0051
3	2	2	1	2	1	1	1	3148.8267	-0.0198
3	2	2	0	2	1	1	0	3148.9814	0.0036
3	1	2	0	2	0	2	1	3226.3387	0.0129
4	2	2	0	3	3	0	1	3232.8452	-0.0149
3	1	2	1	2	0	2	0	3243.7610	-0.0013
3	2	1	0	2	1	1	1	3289.4880	-0.0064
3	2	1	1	2	1	1	0	3307.1133	0.0038
3	2	2	0	2	1	2	1	3405.8973	0.0028
4	1	3	0	3	2	1	1	3415.3553	0.0014
3	2	2	1	2	1	2	0	3423.2559	-0.0244
4	1	3	1	3	2	1	0	3432.6686	-0.0259
4	0	4	1	3	1	3	1	3502.1408	-0.0049
4	1	4	0	3	0	3	0	3534.7528	-0.0116
3	2	1	0	2	1	2	0	3563.9063	-0.0220
3	2	1	1	2	1	2	1	3564.0414	0.0151
4	1	3	1	3	2	2	1	3573.3244	-0.0181
4	1	3	0	3	2	2	0	3573.4849	-0.0007
3	3	1	1	2	2	0	1	3584.6357	-0.0167
3	3	1	0	2	2	0	0	3584.6809	-0.0111
3	3	0	0	2	2	0	1	3585.2972	-0.0071
3	3	0	1	2	2	0	0	3602.9798	0.0062
3	3	1	0	2	2	1	1	3610.8320	0.0182
3	3	1	1	2	2	1	0	3628.4363	-0.0091
3	3	0	1	2	2	1	1	3629.1010	0.0055

3	3	0	0	2	2	1	0	3629.1010	0.0037
4	2	3	1	3	1	2	1	3950.3690	-0.0174
4	2	3	0	3	1	2	0	3950.6473	0.0253
4	2	2	0	3	1	2	1	4299.9833	0.0031
4	2	2	1	3	1	2	0	4317.4241	0.0020
5	2	3	1	4	3	2	1	4348.1680	0.0098
5	2	3	0	4	3	2	0	4348.2514	0.0051
4	1	3	0	3	0	3	1	4352.3508	0.0092
4	1	3	1	3	0	3	0	4369.3955	0.0070
4	3	2	1	3	2	1	1	4454.7664	-0.0206
4	3	2	0	3	2	1	0	4454.9142	0.0005
4	2	3	0	3	1	3	1	4463.9143	0.0112
4	3	1	0	3	2	1	1	4505.4252	-0.0086
4	3	1	1	3	2	1	0	4522.9574	-0.0005
4	3	2	0	3	2	2	1	4595.5704	0.0087
4	3	2	1	3	2	2	0	4612.8994	-0.0194
4	3	1	0	3	2	2	0	4663.5539	-0.0117
4	3	1	1	3	2	2	1	4663.5539	-0.0519
4	4	0	0	3	3	0	1	4847.4379	-0.0133
4	4	1	1	3	3	0	1	4854.1511	0.0114
4	4	1	0	3	3	0	0	4854.1511	-0.0191
4	4	1	0	3	3	1	1	4854.8231	0.0010
4	4	0	1	3	3	0	0	4865.0588	-0.0055
4	4	0	1	3	3	1	1	4865.7271	0.0110
4	4	0	0	3	3	1	0	4865.7271	-0.0057
4	4	1	1	3	3	1	0	4872.4295	0.0083
6	3	3	1	5	4	2	1	4974.3073	0.0166
6	3	3	0	5	4	2	0	4974.3702	0.0073
5	3	3	1	4	2	2	1	5262.7271	-0.0299
5	3	3	0	4	2	2	0	5263.0001	0.0020
4	3	2	0	3	0	3	0	5391.6432	0.0355
4	3	2	1	3	0	3	1	5391.7465	-0.0282
6	2	4	1	5	3	3	1	5422.1625	0.0043
6	2	4	0	5	3	3	0	5422.3000	0.0068
5	3	2	0	4	2	2	1	5446.5393	-0.0128
5	3	2	1	4	2	2	0	5463.9146	-0.0066
5	1	4	1	4	0	4	0	5513.0977	0.0526
7	4	3	1	6	5	2	1	5522.0207	-0.0016
7	4	3	0	6	5	2	0	5522.1464	-0.0067
5	2	4	0	4	1	4	1	5549.2776	-0.0306
5	3	3	0	4	2	3	1	5612.6279	0.0360
5	3	3	1	4	2	3	0	5629.5100	-0.0471
5	4	2	1	4	3	1	1	5764.9278	-0.0150
5	4	2	0	4	3	1	0	5765.0422	-0.0030
5	4	1	0	4	3	1	1	5773.9037	-0.0047
5	4	1	1	4	3	1	0	5791.2844	-0.0068

5	3	2	0	4	2	3	0	5813.3216	-0.0307
5	3	2	1	4	2	3	1	5813.5385	0.0235
5	4	2	0	4	3	2	1	5815.7027	0.0107
5	4	2	1	4	3	2	0	5832.9739	-0.0130
5	4	1	1	4	3	2	1	5841.9441	0.0061
5	4	1	0	4	3	2	0	5841.9441	-0.0085
6	3	4	1	5	2	3	1	6023.3896	-0.0149
6	3	4	0	5	2	3	0	6023.8176	0.0481
5	5	0	0	4	4	0	1	6103.9639	-0.0109
5	5	1	0	4	4	1	1	6105.6546	-0.0048
5	5	1	1	4	4	0	1	6112.3138	-0.0068
5	5	1	0	4	4	0	0	6112.3616	0.0138
5	5	0	1	4	4	1	1	6114.8581	0.0126
5	5	0	0	4	4	1	0	6114.8581	-0.0107
5	5	0	1	4	4	0	0	6121.5581	0.0241
5	5	1	1	4	4	1	0	6123.2490	0.0343
6	4	3	1	5	3	2	1	6613.3733	-0.0225
6	4	3	0	5	3	2	0	6613.6292	0.0119
6	1	5	1	5	0	5	0	6647.5091	0.0383
6	2	5	0	5	1	5	1	6651.9693	-0.0036
6	4	2	0	5	3	2	1	6681.6297	-0.0055
6	4	2	1	5	3	2	0	6698.8108	-0.0111
7	3	5	1	6	2	4	1	6765.8189	0.0409
6	4	3	0	5	3	3	1	6797.4420	0.0295
6	4	3	1	5	3	3	0	6814.2748	-0.0441
6	4	2	0	5	3	3	0	6882.5387	-0.0195
6	4	2	1	5	3	3	1	6882.6264	0.0094
6	5	1	0	5	4	1	1	7042.7935	0.0013
6	5	2	1	5	4	1	1	7046.9997	0.0032
6	5	2	0	5	4	1	0	7047.0782	0.0039
6	5	2	0	5	4	2	1	7056.0492	0.0093
6	5	1	1	5	4	1	0	7060.0234	-0.0045
6	5	1	1	5	4	2	1	7069.0276	0.0341
6	5	1	0	5	4	2	0	7069.0276	-0.0105
6	5	2	1	5	4	2	0	7073.2347	-0.0079
6	3	3	1	5	2	4	1	7098.4031	-0.0056
6	6	0	0	5	5	0	1	7358.1811	-0.0046
6	6	1	0	5	5	1	1	7358.5228	-0.0049
6	6	1	0	5	5	0	0	7366.8760	0.0025
6	6	1	1	5	5	0	1	7366.8760	0.0299
6	6	0	0	5	5	1	0	7367.3546	-0.0173
6	6	0	1	5	5	1	1	7367.3546	0.0091
8	2	6	0	7	3	5	0	7374.9528	-0.0595
6	6	0	1	5	5	0	0	7375.7308	0.0395
6	6	1	1	5	5	1	0	7376.0707	0.0384
7	4	4	1	6	3	3	1	7389.7117	-0.0186

7	4	4	0	6	3	3	0	7390.1427	0.0431
9	0	9	0	8	1	8	0	7746.7983	0.0019
9	1	9	0	8	0	8	0	7746.8291	-0.0188
6	4	3	0	5	1	4	0	7892.1308	-0.0072
7	5	3	1	6	4	2	1	7946.8089	-0.0045
7	5	3	0	6	4	2	0	7947.0014	0.0168
7	5	2	0	6	4	2	1	7962.3500	0.0089
7	5	2	1	6	4	2	0	7979.2672	-0.0112

Table S8. Observed rotational transitions for the thiophenol trimer (Freq) and residuals (o.-c.) according to the Fit of Table 2.

J'	*K'*	*J"*	*K"*	*Freq.* / MHz	*o.-c.* / MHz
5	0	4	0	2330.7050	0.0013
6	0	5	0	2796.8400	0.0043
7	0	6	0	3262.9875	-0.0070
8	0	7	0	3729.1100	0.0047
9	0	8	0	4195.2500	-0.0035
10	0	9	0	4661.3750	0.0007
11	0	10	0	5127.5000	0.0020
12	0	11	0	5593.6250	0.0000
13	0	12	0	6059.7500	-0.0056
14	0	13	0	6525.8500	0.0101
15	0	14	0	6991.9750	-0.0034
16	0	15	0	7458.0875	-0.0088
17	0	16	0	7924.1750	0.0061

Table S9. Calculated tunneling splitting (ΔE_{01}) and barrier height (BH) for the thiophenol dimer at different levels of theory, using the monodimensional torsional Hamiltonian of equation M9.

Method	ΔE_{01} / MHz	BH / cm^{-1}	Effective BH / cm^{-1}
PBEh-3c	13.2	578	439
gCP-B3LYP-D3BJ/def2-TZVP	3.9	689	502
gCP-ωB97X-D3/def2-TZVP	525.5	419	290

Table S10. Atomic coordinates for isomer PD1-trans of the thiophenol dimer according to B2PLYP-D3(BJ)/def2-TZVP.

Atom	*a* / Å[a]	*b* / Å	*c* / Å
C	1.1905	-0.2504	1.2573
C	0.5502	-1.4517	1.5356
C	0.7206	-2.5524	0.7038
C	1.5469	-2.4477	-0.4102
C	2.1951	-1.2524	-0.6952
C	2.0144	-0.1464	0.1371
S	2.8211	1.3642	-0.3188
C	-1.6022	0.9736	-0.1508
C	-1.1444	0.1751	-1.1995
C	-1.6925	-1.0846	-1.4018
C	-2.7092	-1.5549	-0.5768
C	-3.1713	-0.7539	0.4619
C	-2.6196	0.5031	0.6805
S	-0.9443	2.5852	0.1697
H	1.0277	0.6079	1.8948
H	-0.0980	-1.5182	2.3990
H	0.2109	-3.4813	0.9194
H	1.6912	-3.2991	-1.0622
H	2.8428	-1.1782	-1.5595
H	2.5787	2.0228	0.8215
H	-0.3546	0.5312	-1.8469
H	-1.3182	-1.7008	-2.2084
H	-3.1347	-2.5356	-0.7405
H	-3.9607	-1.1082	1.1119
H	-2.9721	1.1156	1.5006
H	0.1661	2.4486	-0.5699

[a]Principal inertial axes denoted *a*, *b*, *c*.

Table S11. Atomic coordinates for isomer PD1-cis of the thiophenol dimer according to B2PLYP-D3(BJ)/def2-TZVP.

Atom	*a* / Å[a]	*b* / Å	*c* / Å
C	-2.6409	-0.4361	-0.6584
C	-1.6207	-0.9502	0.1434
C	-1.1207	-0.1836	1.1967
C	-1.6283	1.0874	1.4317
C	-2.6476	1.6007	0.6360
C	-3.1529	0.8314	-0.4065
S	-1.0139	-2.5718	-0.2222
C	1.2276	0.1961	-1.2436
C	0.5915	1.3846	-1.5774
C	0.7514	2.5169	-0.7865
C	1.5587	2.4535	0.3440
C	2.1924	1.2666	0.6923
C	2.0248	0.1309	-0.1002
S	2.7597	-1.4307	0.3002
H	1.0863	-0.6849	-1.8555
H	-0.0402	1.4191	-2.4547
H	0.2481	3.4380	-1.0459
H	1.6935	3.3289	0.9658
H	2.8108	1.2262	1.5797
H	3.4218	-1.0206	1.3894
H	-0.3291	-0.5740	1.8218
H	-1.2209	1.6785	2.2410
H	-3.0416	2.5900	0.8251
H	-3.9447	1.2191	-1.0342
H	-3.0256	-1.0230	-1.4827
H	0.1202	-2.4788	0.4872

[a]Principal inertial axes denoted *a*, *b*, *c*.

Table S12. Atomic coordinates for isomer PD2-trans of the thiophenol dimer according to B2PLYP-D3(BJ)/def2-TZVP.

Atom	*a* / Å[a]	*b* / Å	*c* / Å
C	-2.0483	-0.0871	0.9345
C	-1.9982	1.1260	1.6103
C	-1.5902	2.2826	0.9540
C	-1.2367	2.2204	-0.3896
C	-1.3014	1.0159	-1.0781
C	-1.7078	-0.1427	-0.4167
S	-1.7101	-1.6613	-1.3325
C	1.5221	-0.6381	0.4305
C	1.7878	-0.7499	-0.9361
C	2.2020	0.3638	-1.6558
C	2.3789	1.5890	-1.0193
C	2.1269	1.6954	0.3438
C	1.6888	0.5917	1.0665
S	1.0235	-2.0349	1.4012
H	-2.3358	-0.9871	1.4605
H	-2.2697	1.1616	2.6571
H	-1.5454	3.2232	1.4862
H	-0.9122	3.1120	-0.9090
H	-1.0360	0.9736	-2.1258
H	-2.6097	-2.3029	-0.5753
H	1.6745	-1.7052	-1.4300
H	2.3980	0.2692	-2.7161
H	2.7107	2.4516	-1.5813
H	2.2523	2.6451	0.8471
H	1.4591	0.6856	2.1193
H	0.2782	-2.6087	0.4439

[a]Principal inertial axes denoted *a*, *b*, *c*.

Table S13. Atomic coordinates for isomer PD2-cis of the thiophenol dimer according to B2PLYP-D3(BJ)/def2-TZVP.

Atom	*a* / Å[a]	*b* / Å	*c* / Å
C	1.5972	0.7103	1.0700
C	1.9546	1.8414	0.3450
C	2.2588	1.7425	-1.0078
C	2.2185	0.4981	-1.6310
C	1.8877	-0.6404	-0.9069
C	1.5654	-0.5372	0.4481
S	1.1676	-1.9641	1.4230
C	-1.6577	-0.2395	-0.4587
C	-2.0354	-0.2588	0.8843
C	-2.1155	0.9303	1.5983
C	-1.8129	2.1419	0.9859
C	-1.4307	2.1563	-0.3514
C	-1.3567	0.9741	-1.0762
S	-1.6003	-1.7843	-1.3225
H	1.3242	0.7949	2.1131
H	1.9749	2.8046	0.8374
H	2.5272	2.6253	-1.5723
H	2.4624	0.4096	-2.6820
H	1.8885	-1.6103	-1.3857
H	0.4519	-2.5871	0.4740
H	-2.2506	-1.1998	1.3725
H	-2.4071	0.9049	2.6399
H	-1.8704	3.0652	1.5462
H	-1.1840	3.0912	-0.8368
H	-1.0570	0.9967	-2.1151
H	-0.9116	-1.3434	-2.3827

[a]Principal inertial axes denoted *a*, *b*, *c*.

Table S14. Hydrogen bond distances for the dimers of hydrogen sulfide and water.

Dimer	Hydrogen bond	Method	*r*(X···Y) / Å[a]	*r*(X-H···Y) / Å	∠(X-H···Y) / deg
$(H_2O)_2$	O-H···O	MW[b]	2.980(10)		
		MW[c]	2.976		
		IR[d]	2.952		
		MP2[e]		1.951	171
$(H_2S)(H_2O)$	O-H···S	MW[f]	3.535(3)	2.597(4)	193.4(4)
		MW[g]	3.544(8)	2.590(6)	185.2(63)
		MP2[h]	3.480	2.52	187.8
$(H_2O)(H_2S)$	S-H···O	MW[i]	3.55	2.195	180
$(H_2S)_2$	S-H···S	Experiment[e]	4.112(1)	2.778(9)	175(7)
		MP2/aug-cc-pVDZ[e]	4.096	2.743	173

[a]X, Y denotes O or S atoms. [b]R. Dyke, J. S. Muenter, *J. Chem. Phys*. **1974**, *60*, 2929-2930. T. R. Dyke, K. M. Mack, J. S. Muenter, *J. Chem. Phys*. **1977**, *66*, 498-510. [c]J. A. Odutola, T.R. Dyke, *J. Chem. Phys*. **1980**, *72*, 5062-5070. [d]A. Mukhopadhyay, W. T.S. Cole, R. J. Saykally, *Chem. Phys. Lett*. **2015**, *633*, 13–26. [e]MP2/aug-cc-pVDZ: A. Das, P. K. Mandal, F. J. Lovas, C. Medcraft, N. R. Walker, E. Arunan, *Angew. Chem. Int. Ed*. **2018**, *57*, 15199-15203. [f]A_2 state: F. Lovas, private communication, 2020. [g]B_2 state: F. Lovas, private communication, 2020. [h]MP2/6- 311++G(3d1f,3p1d): Y.-B. Wang, F.-M. Tao, Y.-K. Pan, *Chem. Phys. Lett.* **1994**, *230*, 480-484. [i]F. Lovas, private communication, 2020.

Table S15. Atomic coordinates for isomer UUU of the thiophenol trimer according to B2PLYP-D3(BJ)/def2-TZVP.

Atom	*a* / Å[a]	*b* / Å	*c* / Å
C	3.1416	-1.3932	-0.2141
C	2.4717	-0.1903	-0.4519
C	1.9976	0.5607	0.6243
C	2.1809	0.0996	1.9223
C	2.8490	-1.0970	2.1629
C	3.3334	-1.8380	1.0890
H	3.5018	-1.9831	-1.0473
S	2.2732	0.3234	-2.1364
H	1.4826	1.4952	0.4541
H	1.7960	0.6862	2.7457
H	2.9930	-1.4485	3.1757
H	3.8523	-2.7716	1.2616
H	1.2255	1.1413	-1.9467
C	-0.4998	-2.0117	0.6203
C	-1.4098	-2.0423	-0.4372
C	-2.7811	-2.0211	-0.1702
C	-3.2347	-1.9695	1.1429
C	-2.3282	-1.9247	2.1981
C	-0.9633	-1.9442	1.9286
H	0.5632	-2.0336	0.4275
S	-0.9014	-2.1231	-2.1332
H	-3.4894	-2.0339	-0.9888
H	-4.2988	-1.9521	1.3381
H	-2.6831	-1.8771	3.2188
H	-0.2456	-1.9073	2.7372
H	0.3283	-1.6086	-1.9716
C	-0.3610	3.4169	-0.1612
C	-1.0740	2.2428	-0.4167
C	-1.4821	1.4374	0.6474
C	-1.1632	1.8002	1.9503
C	-0.4553	2.9700	2.2084
C	-0.0611	3.7794	1.1471
H	-0.0338	4.0384	-0.9850
S	-1.4369	1.8497	-2.1064
H	-2.0396	0.5301	0.4637
H	-1.4746	1.1587	2.7637
H	-0.2145	3.2500	3.2251
H	0.4925	4.6903	1.3333
H	-1.6028	0.5268	-1.9461

[a]Principal inertial axes denoted *a*, *b*, *c*.

Table S16. Results from a (second-order intramonomer / third-order intermonomer) Symmetry-Adapted Perturbation Theory (SAPT2+(3)/aug-cc-pVDZ) binding energy decomposition of $(thiophenol)_2$ and related dimers, comparing the magnitude of the electrostatic and dispersion contributions (all values in kJ mol^{-1}).

	$\Delta E_{Electrostatic}$	$\Delta E_{Dispersion}$	ΔE_{Ind}	ΔE_{Exch}	ΔE_{Total}	ΔE_{S22}[f]
$(H_2O)_2$ [a]	-35.7[191.9%]	-9.5[50.8%]	-11.1[59.7%]	37.7	-18.6	-21.0
$(Phenol)_2$ [b]	-41.8[151.3%]	-28.8[104.3%]	-15.9[57.6%]	58.9	-27.6	-29.5
$(Aniline)_2$ [c]	-24.6[75.6%]	-39.3[120.6%]	-6.9[21.1%]	38.2	-32.6	
$(H_2S)_2$ [d]	-12.1[223.7%]	-7.8[144.5%]	-4.7[87.0%]	19.2	-5.4	
$(Thiophenol)_2$ PD1-trans[e]	-24.9[96.4%]	-47.9[185.2%]	-7.7[29.7%]	54.6	-25.9	
$(Thiophenol)_2$ PD2-cis[e]	-26.2[97.4%]	-53.3[198.5%]	-8.4[31.2%]	61.0	-26.9	
Pyridine-CH_4 [f]	-3.0[57.5%]	-10.9[208.1%]	-0.7[13.5%]	9.4	-5.2	

[a]T. R. Dyke, K. M. Mack, J. S. Muenter, *J. Chem. Phys.*, **1977**, *66*, 498–510. [b]N. A. Seifert, A. L. Steber, J. L. Neill, C. Pérez, D. P. Zaleski, B. H. Pate, A. Lesarri, *Phys. Chem. Chem. Phys.* **2013**, *15*, 11468-11477. [c]A. Das [d]A. Das, P. K. Mandal, F. J. Lovas, C. Medcraft, N. R. Walker, E. Arunan, *Angew. Chem. Int. Ed.*, **2018**, *57*, 15199–15203. [e]This work. [f]Q. Gou, L. Spada, M. Vallejo-López, A. Lesarri, E. J. Cocinero, W. Caminati, *Phys. Chem. Chem. Phys.*, **2014**, *16*, 13041–13046. [f]Interaction energies in Hobza's S22 database, recalculated by Sherrill: T. Takatani, E. G. Hohenstein, M. Malagoli, M. S. Marshall, C. D. Sherrill, *J. Chem. Phys.*, **2010**, *132*, 144104.
